# Supplementary material for: Periodic synchronisation of dengue epidemics in Thailand over the last 5 decades driven by temperature and immunity
Source: PLoS Biol. 2022 Mar 18;20(3):e3001160. doi: 10.1371/journal.pbio.3001160 (PMC8967062; doi:10.1371/journal.pbio.3001160)
Supplement: S1 Appendix — (PDF) [file pbio.3001160.s001.pdf]

Supporting Information for "Periodic synchronization of dengue epidemics in Thailand over the last five decades driven by temperature and immunity"

Bernardo García-Carreras*, Bingyi Yang, Mary K. Grabowski, Lawrence W. Sheppard, Angkana T. Huang, Henrik Salje, Hannah Eleanor Clapham, Sopon Iamsirithaworn, Pawinee Doung-Ngern, Justin Lessler, Derek A. T. Cummings

* bgarciacarreras@gmail.com

Contents

A note on estimating significance of (cross-)wavelet mean fields 2

Caveats on the dengue model 2

Further details on simulation studies 3

Robustness tests 3

Perspectives on synchrony 9

Weighted median timescales . . . 9
Moving window spline correlograms . . . 11
Wavelet mean fields . . . 12
Weighted median phase angles . . . 12
Delays in peaks and nadirs . . . 14

Additional simulation 1 results 15

Additional simulations 2 and 3 results 22

Additional results on synchrony in temperature 37

Patterns in temperature across Thailand 39

A note on estimating significance of (cross-)wavelet mean fields

We were specifically interested in knowing at which points in time, rather than at what multiannual timescales, synchrony was significant. This led us to the multi-step approach to estimating significance outlined in the main text, where we make comparisons based on slices in time averaged across timescales. However, we estimate significance of (cross-)wavelet mean fields using a different quantity, the (cross-)wavelet phasor mean field (see section "Materials and methods", subsection "Wavelet mean fields as a measure of synchrony" in the main text). The reason derives from the choice of working with slices in time. The WMFs are normalised so that the time average (RMS) at any timescale has a maximum value of 1, if the wavelet components drawn from each location have matching phase and amplitude dynamics. This normalisation allows comparison of timescale-dependent time average synchrony values, and straightforward significance testing of the time averages. However, when averaging across timescales, the WMF values at a given time are not bounded in this way, as all the wavelet amplitudes at a particular time may be much higher than their time average. Times when wavelet amplitudes are above average are likely to have high WMF values whether or not the transforms from different locations are in phase, potentially implying a different null hypothesis at each time and/or introducing ambiguity about the meaning of surrogate testing results. Instead we elected to use significance testing of the phase information only, to pick out times when the transforms were in phase, associated with features appearing in both the WPMF and WMF. The WPMFs only retain information on the phases, and are thus not affected by the issues around amplitude normalisation. The same statistical test can be applied across times and timescales to identify times of high synchrony. In general, these times are also associated with high wavelet amplitudes, enhancing the prominence of the synchronised features as they appear in the WMF.

Caveats on the dengue model

The temperature dependent traits included in the model leverage and synthesise the results of laboratory experiments, and allow temperature, and its strongly nonlinear effects on dengue transmission, to be included in the model. However, a number of simplifying assumptions are also made, to include:

1. The temperature dependence of some traits are the result of fitting to relatively few experimental data points (e.g., transmission probability; [1]). The (low and high) temperatures at which a trait is reduced to zero are also the temperatures at which it is notoriously difficult to perform experiments, and so both the lower and upper ranges tend to be less well defined.

2. The model assumes the temperature dependence of these traits are time-invariant, and ignore the potential for seasonal variation as well as acclimation and evolution [2].

3. Experiments are typically performed on specific mosquito strains (so genetically more homogeneous), thus potentially substantially underestimating the variability in these traits across individuals.

4. These experiments isolate single mechanisms to the extent possible, but potentially overlook complex interdependencies between these traits, and necessarily simplify mechanisms that can be more complex (e.g., [3]).

5. The population dynamics of mosquitoes are also substantially simplified, omitting, for instance, age and stage classes that could be important to characterise their dynamics. For some aspects of the model, no data is available. For example, the expression for the carrying capacity was adapted by Huber *et al.* [4] from Palamara *et al.* [5], a study on *Paramecium caudatum*, so while temperature dependence of carrying capacity may seem a priori reasonable, we are yet to more carefully understand how it manifests (if at all) in mosquito species (see section "Robustness tests").

6. Experiments on which these functions rely were performed under constant temperature conditions, a situation rarely encountered in reality; accounting for rapidly fluctuating temperatures would

probably alter the shape of these temperature functions [6–8]. For example, accounting for field conditions could dramatically increase the mortality rates of mosquitoes [8] (see section "Robustness tests"). We also assume traits change instantaneously with temperature; in fact, there may be lags.

7. For some analyses, we run the model using temperature time series for Thai provinces. These time series are taken from a gridded temperature dataset. Its spatio-temporal resolution is very coarse, and estimates are based on temperature being measured at heights that are likely very different to where mosquitoes live [9]. Furthermore, a location might present a wide range of microclimates, and as a result, the true temperature experienced by a mosquito might differ significantly from the mean temperature given in the gridded dataset.

8. Although Mordecai *et al.* [1] provide estimates of uncertainty around their fits, we do not leverage that information in our simulations; we here only use the parameter point estimates.

Further details on simulation studies

In the simulations described in Table 1 in the main text, each location was run as an independent simulation (no host movement between locations), using the same host birth and death rates and starting number of infecteds (see section "Temperature-dependent dengue model" in the main text).

For simulation 1, total populations sizes were those of the corresponding provinces, and we ran the models for 497 years by concatenating seven 71-year temperature time series (1948–2018) for each province, to allow the dengue dynamics to settle. We then analysed the total number of infected individuals across all four serotypes for the last 51 years of the simulation.

For simulations 2 and 3, we chose six hypothetical locations each with a population size of $1 \cdot 10^6$, characterised by temperatures that went from a higher mean and lower variability, to lower mean and higher variability, roughly corresponding to the patterns observed in Thailand (Figs S40–S42). Each of the six locations was run independently for 500 years, of which the last 51 were analysed.

In simulation 3, we introduce a series of single multiannual fluctuations, at the same time across locations. Because different thermal regimes produce a range of dengue dynamics, these multiannual fluctuations might take place at different points in their immune dynamics (or take place at different phases). For this reason, all simulations were repeated starting the multiannual fluctuation at four different times of year, and on ten consecutive years. Results were insensitive to when a multiannual fluctuation took place.

Robustness tests

We checked the robustness of our results to three of the assumptions made in the model, and did so by repeating two sets of simulations: (i) using real temperature time series across provinces in Thailand (simulation 1; Table 1 in the main text); and (ii) using synthetic, sinusoidal time series across six hypothetical locations, where a single multiannual fluctuation is introduced across all locations (simulation 3; as in Fig 4 in the main text).

First, we varied the temperature dependence of carrying capacity (E_{a}) of the mosquito population. The reason for focussing on this parameter is that the actual value used in Huber *et al.* [4] is ambiguous (the values in the main text and supplementary code are different), and because the value of E_{a} has, in any case, little theoretical or empirical support in the literature. Throughout our study, we assumed $E_{\mathrm{a}} = 0.05$; we also ran simulations with two additional values of E_{a}: 0, and 0.5 (i.e., no temperature dependence in carrying capacity, and a stronger temperature dependence than that used in the main text, respectively). While the dynamics produced do differ as a function of the value of E_{a} (peaks and troughs tend to be accentuated as E_{a} increases), the oscillatory behaviour remains very similar (Figs S1 and S2).

Second, we used an alternative formulation for the temperature-dependent mortality rate of *Aedes aegypti*. Specifically, we used the field longevity of mosquitoes in Johansson *et al.* [10],

$$\mu^{\mathrm{v}} = 3.966922 \cdot 10^{-1} - 3.912447 \cdot 10^{-2}\,T + 2.422395 \cdot 10^{-3}\,T^2 - \\ 7.479176 \cdot 10^{-5}\,T^3 + 9.297834 \cdot 10^{-7}\,T^4. \tag{1}$$

It is important to note that this mortality rate is almost four times higher than that otherwise used elsewhere in this study (as it corresponds to field conditions, as opposed to laboratory). Making this single alteration to the model is naive, inasmuch as all other temperature-dependent rates are likely to differ (possibly) significantly in field conditions. Nonetheless, although dynamics of dengue do change between the two different formulations of mortality, our results on synchrony remain similar (Figs S3 and S4), although in the simulation 3 results, synchrony is constrained across a narrower range of timescales when using the lower mosquito mortality rate (Fig S4).

Third, we used an alternative value of M (the ratio between mosquitoes and hosts). The degree of transmission and R_0 are proportional to M (see Fig S44). In the main text we justify our choice of $M = 1.5$ by arguing that within the temperature ranges observed in Thailand this led to values of R_0 consistent with those reported for dengue in Thailand. Nonetheless, we also ran the model with lower and higher values of M, and our results are robust to these changes (Figs S5 and S6 show results for $M = 1$).

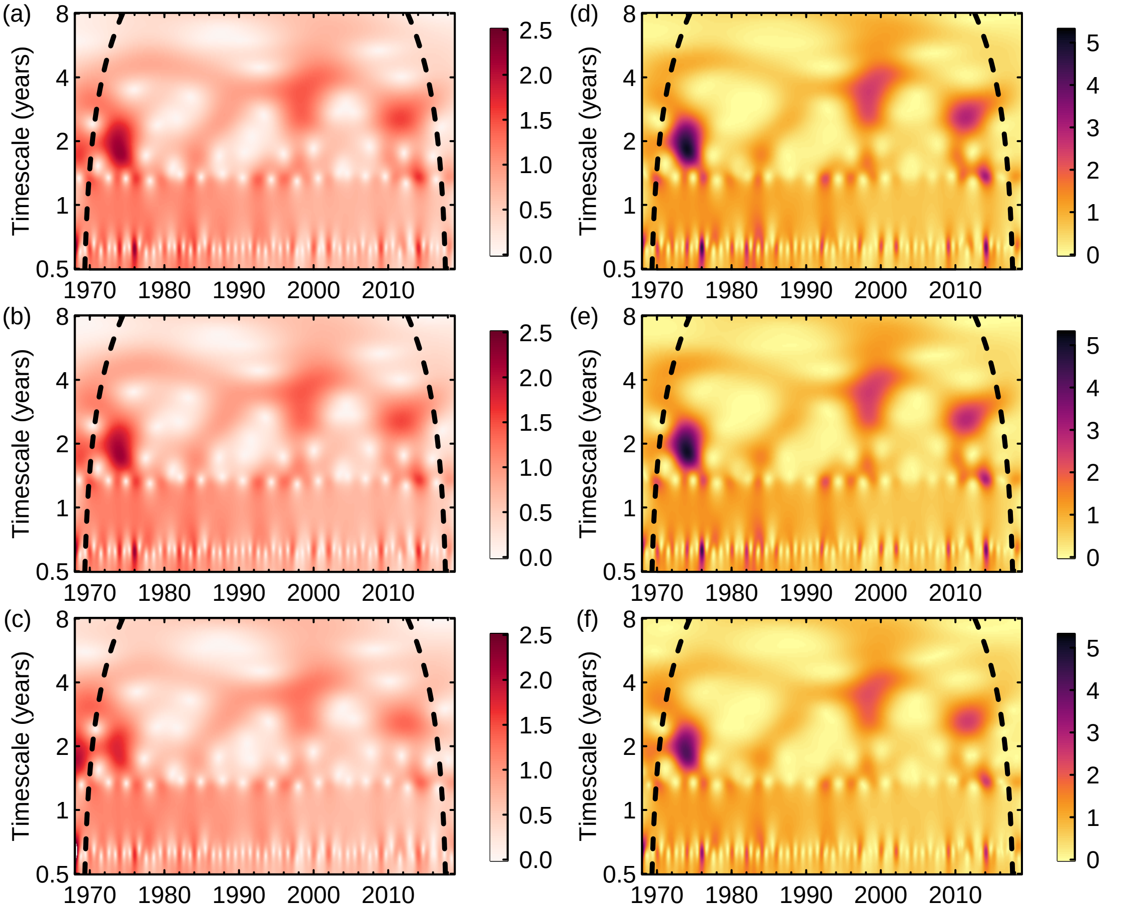

Fig S1. (Cross-)wavelet mean fields for different temperature dependences of carrying capacity, using real temperature time series. (a–c) Wavelet mean fields, and (d–f) cross-wavelet mean fields for simulations using real temperature time series (simulation 1), using temperature dependence of carrying capacity values (a,d) $E_a = 0$, (b,e) $E_a = 0.05$ (thus analogous to Fig 2c,e in the main text), and (c,f) $E_a = 0.5$. Here, mean cross-protection is assumed to be one year. The underlying data are in S2 Data at `https://github.com/UF-IDD/synchrony_dengue_figures`.

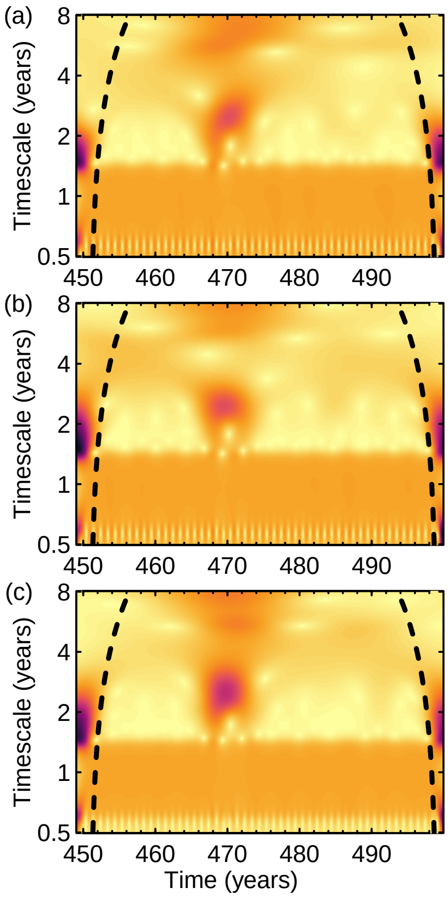

Fig S2. Wavelet mean fields for different temperature dependences of carrying capacity, across the six hypothetical locations. (a–c) Wavelet mean fields for simulations using synthetic temperature time series across six hypothetical locations, with a single four-year multiannual fluctuation (simulation 3), using temperature dependence of carrying capacity values (a) $E_{\mathrm{a}} = 0$, (b) $E_{\mathrm{a}} = 0.05$ (thus analogous to Fig 4e in the main text), and (c) $E_{\mathrm{a}} = 0.5$. Here, we assume mean cross-protection to be one year, and the amplitude of the multiannual fluctuation is 0.2 times that of the seasonal cycle. The underlying data are in S2 Data at `https://github.com/UF-IDD/synchrony_dengue_figures`.

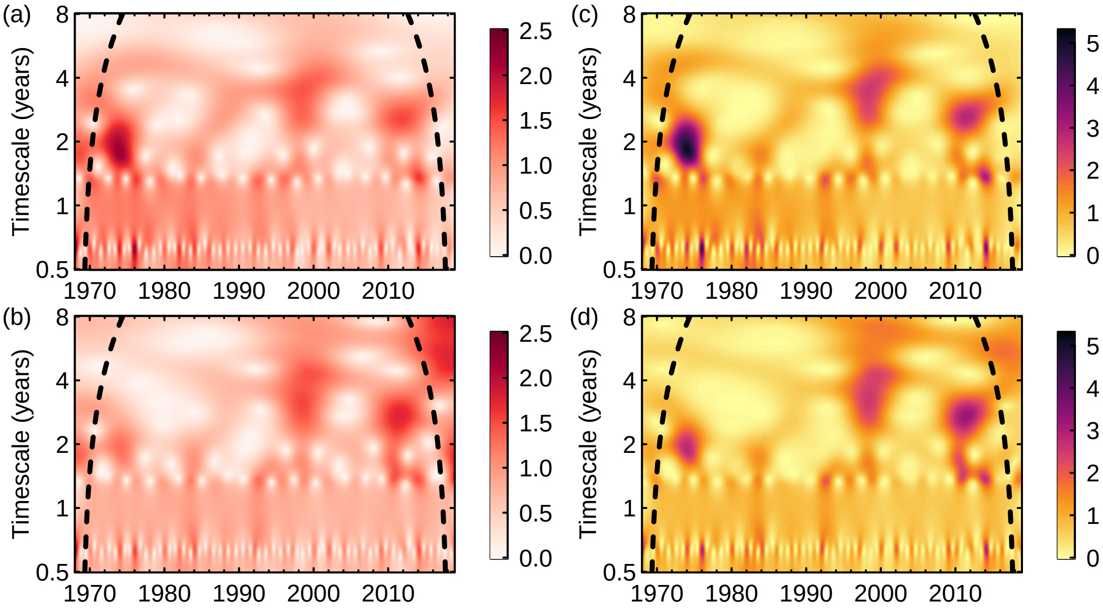

Fig S3. (Cross-)wavelet mean fields for an alternative formulation of mosquito mortality rate, using real temperature time series. (a,b) Wavelet mean fields, and (c,d) cross-wavelet mean fields simulations using real temperature time series (simulation 1) for the temperature-dependent mortality rate in (a,c) Mordecai *et al.* and Huber *et al.* [1,4], and (b,d) the temperature-dependent field mortality rate in Johansson *et al.* [10]. Panels (a,c) are the same as Fig 2c,e in the main text. Here, we assume mean cross-protection to be one year. The underlying data are in S2 Data at `https://github.com/UF-IDD/synchrony_dengue_figures`.

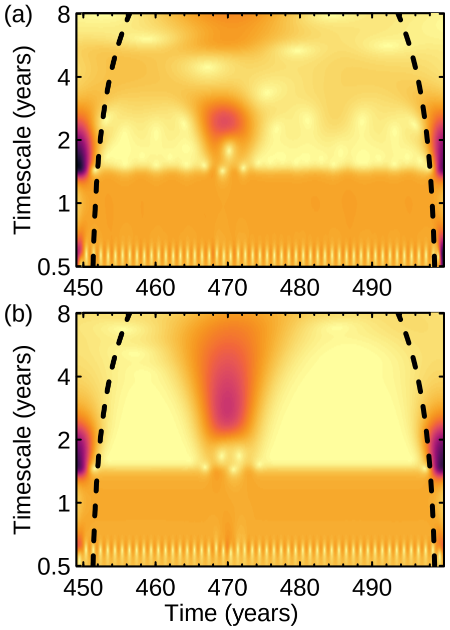

Fig S4. Wavelet mean fields for different formulations of mosquito mortality rate, across the six hypothetical locations. (a,b) Wavelet mean fields for simulations using synthetic temperature time series across six hypothetical locations, with a single four-year multiannual fluctuation (simulation 3), for the temperature-dependent mortality rate in (a) Mordecai *et al.* and Huber *et al.* [1,4] used in this study, and (b) the temperature-dependent field mortality rate in Johansson *et al.* [10]. Here, we assume mean cross-protection is one year, and the amplitude of the multiannual fluctuation is 0.2 times that of the seasonal cycle. The underlying data are in S2 Data at `https://github.com/UF-IDD/synchrony_dengue_figures`.

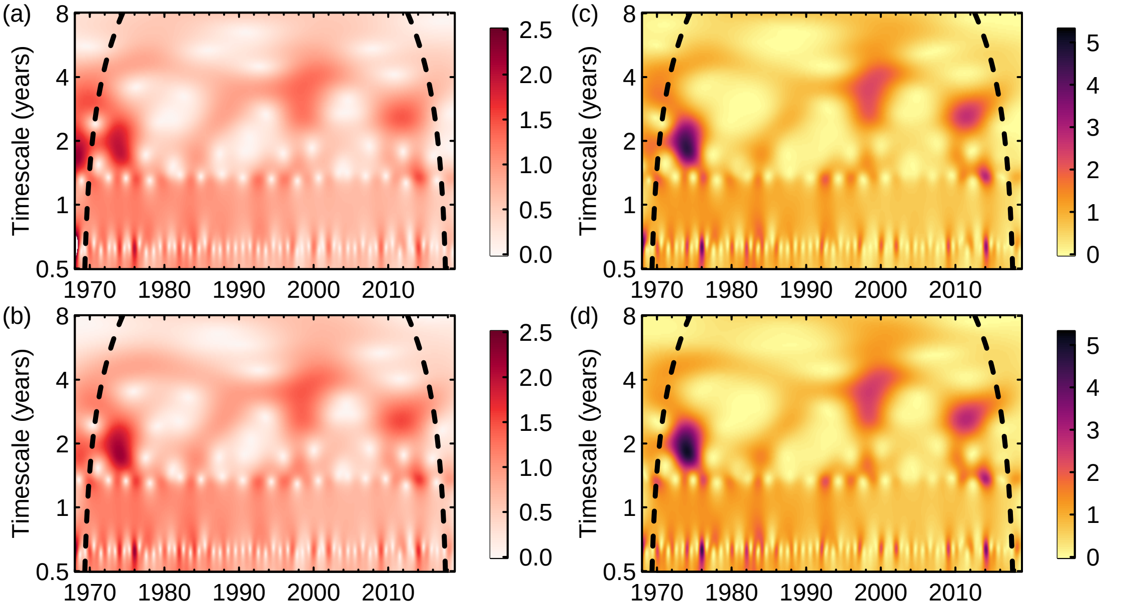

Fig S5. (Cross-)wavelet mean fields for different ratios of mosquitoes to humans M, using real temperature time series. (a,b) Wavelet mean fields, and (c,d) cross-wavelet mean fields simulations using real temperature time series (simulation 1) for (a,c) $M = 1$, and (b,d) $M = 1.5$ (used in this study; see Fig S44 to see how R_0 values differ when using these two values of M). Panels (b,d) are the same as Fig 2c,e in the main text. Here, we assume mean cross-protection to be one year. The underlying data are in S3 Data at `https://github.com/UF-IDD/synchrony_dengue_figures`.

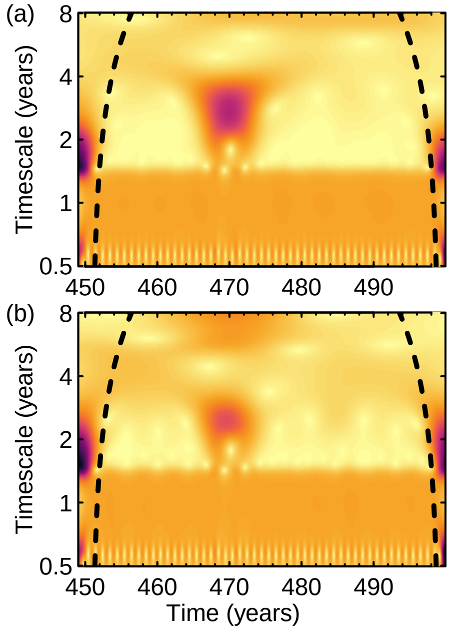

Fig S6. Wavelet mean fields for different ratios of mosquitoes to humans M, across the six hypothetical locations. (a,b) Wavelet mean fields for simulations using synthetic temperature time series across six hypothetical locations, with a single four-year multiannual fluctuation (simulation 3), for (a) $M = 1$, and (b) $M = 1.5$ (used in this study). M is proportional to R_0 (see Fig S44). Here, we assume mean cross-protection is one year, and the amplitude of the multiannual fluctuation is 0.2 times that of the seasonal cycle. The underlying data are in S3 Data at `https://github.com/UF-IDD/synchrony_dengue_figures`.

Perspectives on synchrony

In this section, we provide details, explanations, and results for the four additional different perspectives on synchrony shown in Fig 3 in the main text (wavelet mean fields are presented in the main text).

Weighted median timescales

The weighted median timescale describes what multiannual timescales dominate a signal as a function of time. More specifically, it is defined as the median timescale (between 1.5–5 years) weighted by the corresponding wavelet power at each scale, for each point in time (e.g., thick red line in Fig S7). We also estimate the weighted interquantile range, defined as the width of the 75% interquantile range, weighted by wavelet power. We use this as a measure of confidence in the weighted median timescale: a large value suggests power is widely distributed across timescales while smaller values mean that power is more narrowly distributed around the median. We use function 'wtd.quantile' in R package 'Hmisc' v4.2.0 to estimate these statistics.

In moments of greater spatial synchrony (in which fluctuations across different locations are more similar), we might expect the distributions of wavelet power across locations to be more similar, and the dominant multiannual timescales across locations to be more tightly distributed. Similarly, during periods of greater asynchrony, we would expect dominant multiannual timescales to be less tightly distributed. To this end, we estimated weighted median absolute deviations (MADs; where $\text{MAD} = \text{weighted median}(|p_i - \text{weighted median}(p)|)$) of the weighted median timescales for each point in time, across provinces. In calculating the MADs, we used the inverse of the weighted interquantile range as weighting. Lower values of MAD (tighter distributions) correspond to greater synchrony.

The MADs shown in Fig S8c describe a system where the degree of synchrony itself appears to oscillate; the country moves in and out of synchrony.

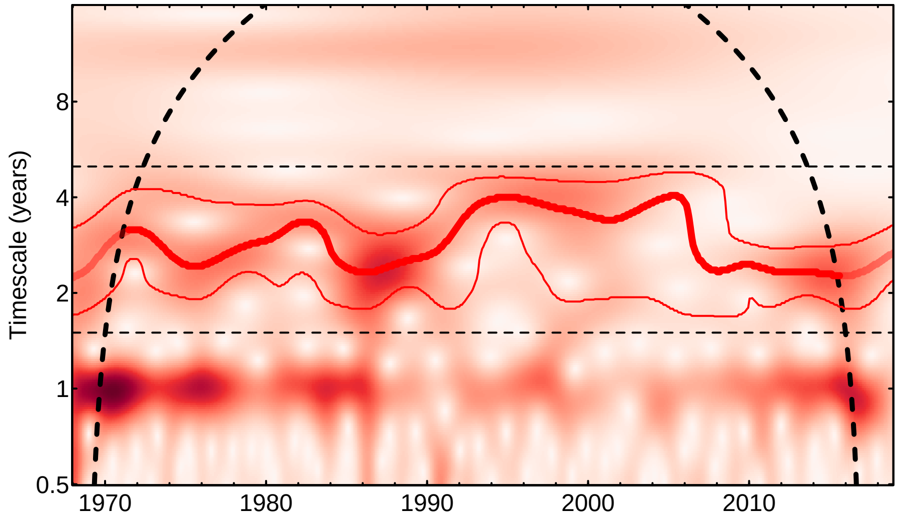

Fig S7. Wavelet power for dengue cases in Bangkok. Wavelet power for ln-dengue cases in Bangkok (here shown as square-root of power for clarity). Darker reds indicate greater wavelet power. The thick red line shows the weighted median timescales for Bangkok (this line corresponds to the red line in Fig S8b). Thin red lines show the weighted interquantile range, used to weight the weighted median timescales across provinces (see section "Materials and methods"). Horizontal dashed lines show the chosen multiannual range (1.5–5 years). Note that the 1.5 year lower limit excludes the annual component and prevents any leakages into the multiannual range (also confirmed across other provinces). The underlying data are in S3 Data at `https://github.com/UF-IDD/synchrony_dengue_figures`.

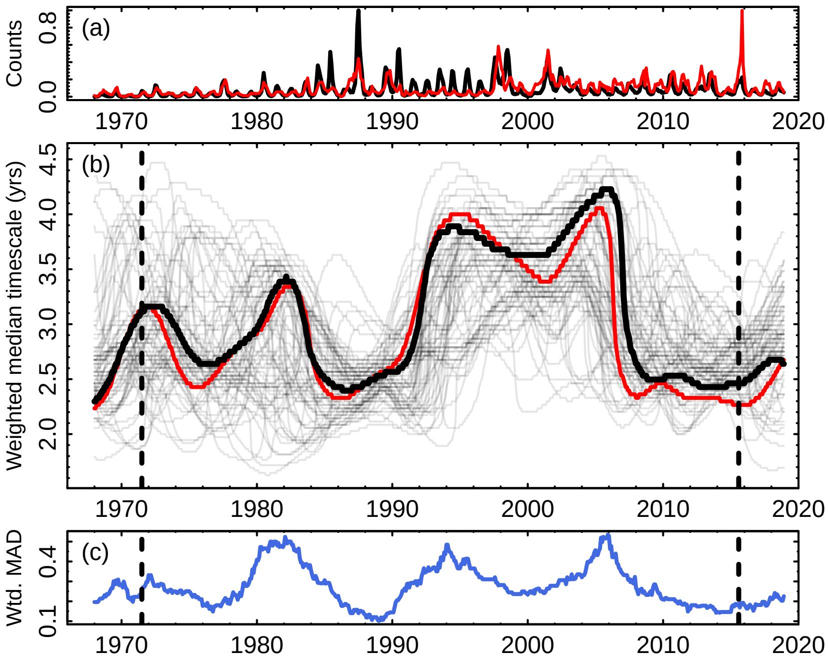

Fig S8. Spatial synchrony using weighted median timescales. (a) Total dengue cases across Thailand, and for Bangkok, normalised by the maximum number of cases in each time series for clarity. (b) Weighted median timescale, indicating the dominant multiannual timescale for each province at each point in time (see Fig S7). (c) The median absolute deviation (MADs), estimated on the time series shown in panel (b), which measures the relative dispersion of the weighted median timescales across provinces at each point in time, while accounting for their uncertainty. Each gray line corresponds to a province, Bangkok is highlighted in red (the red line is the same as that in Fig S7), and black corresponds to the estimate for the total counts across all provinces. Periods during which the weighted MADs in (c) are lower correspond to higher degrees of synchrony. Edge effects in the WTs may influence results before and after the vertical dashed lines. The underlying data are in S3 Data at `https://github.com/UF-IDD/synchrony_dengue_figures`.

Moving window spline correlograms

To better understand the spatial structure of synchrony, we estimate spline correlograms (function 'spline.correlog' in R package 'ncf' v1.2.8; [11, 12]). The seasonal cycles are highly correlated throughout Thailand, as would be expected. To focus on correlations in the multiannual components only, we applied the spline correlogram methods on the time series reconstructions from the WT using multiannual timescales only (having therefore also removed the seasonal component; Fig 1b in the main text). Spline correlograms describe how correlations between reconstructed time series of pairs of provinces decay as a function of the distance between them (they measure spatial autocorrelation), and provide an indication for the average distances below which Pearson correlations are higher than would be expected between two provinces at random (i.e., above the country-wide correlation). We calculate these spline correlograms for a five-year moving window such that windows included at least a single multiannual cycle, thus providing estimates for how correlations change over time. Results describe a system where incidence fluctuates in and out of synchrony (Fig S9). During periods of greater synchrony, correlations are high and almost independent of distance (i.e., synchrony extends across the entire country).

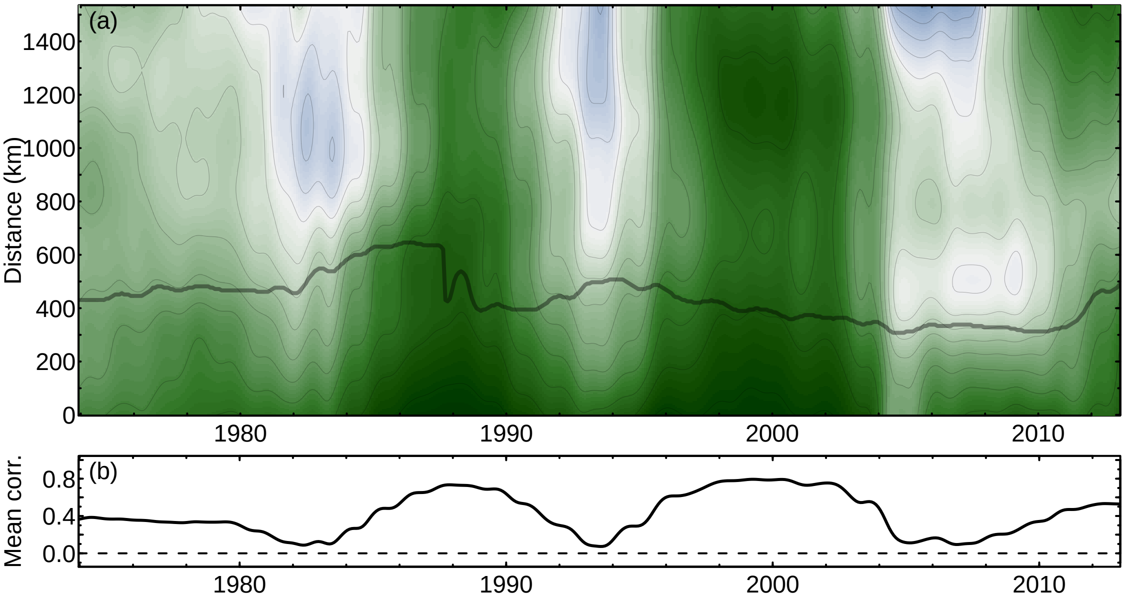

Fig S9. Synchrony using spline correlograms. (a) Spline correlograms for a five-year moving window (with time on the x-axis and pairwise distances between Thai provinces on the y-axis). The colour indicates the pairwise correlation in reconstructions of time series using multiannual components only. Green, white, and blue are positive, zero, and negative correlations, respectively. The single thick grey line shows the distance at each point in time for which the pairwise correlation is no greater than could be expected at random between any two provinces. (b) Average correlation across all distances within the five-year window, for each point in time. The underlying data are in S3 Data at `https://github.com/UF-IDD/synchrony_dengue_figures`.

Wavelet mean fields

Wavelet mean fields are detailed in section “Materials and methods” in the main text, and results are shown in Fig 2b in the main text.

Weighted median phase angles

During periods of greater synchrony, we might expect the phases in different provinces to be more similar, and phase angles between provinces might be expected to be more narrowly distributed. For instance, in a period of greater synchrony, we might expect the phases of provinces to be more similar, and the phase angles between all provinces to be smaller on average, relative to periods of less synchrony. For each province at a time, we estimate the cross-wavelet between it and all other provinces (thus, with a total of 72 provinces, a total of 71 comparisons). The cross-wavelet contains information on the phase angle between the two time series, for each s and τ. We calculate the absolute median phase angle weighted by cross-wavelet power for each point in time, across the range of timescales of interest, yielding 71 time series of weighted absolute median phase angles. We also extract the weighted interquantile range as a measure of confidence in the median values. Then, for each point in time, we estimate a median phase angle across the (71) pairwise comparisons, weighted by the inverse of the interquantile range, producing a single time series of weighted median phase angles for that province. This is repeated for each province, producing a time series of median absolute phase angles associated with each province, the overall phase angles (Fig S10d). Finally, for each point in time, we calculated the median absolute deviation (MAD) across the 72 provinces (Fig S10e), using the inverse of the weighted interquantile range as weighting. The phase angle MAD estimates the spread of phase angles across provinces; when this value is smaller, it suggests that outbreaks are happening more synchronously across the country.

The phases at timescales of two and four years (Fig S10b,c) confirm that synchrony takes places at different timescales. In the 2-year phases, there appears to be greater synchrony (there is greater overlap in lines) in the latter half of the 1980s, and to a lesser extent in the mid-2010s, while in the 4-year phases, synchrony is greater between the mid-1990s and early 2000s. These observations confirm the patterns obtained using WMFs (Figs 2b and 3 in the main text). The measure of synchrony obtained from these phase angles (Fig S10e) is qualitatively very similar to that obtained from the weighted median timescales (Fig S8c).

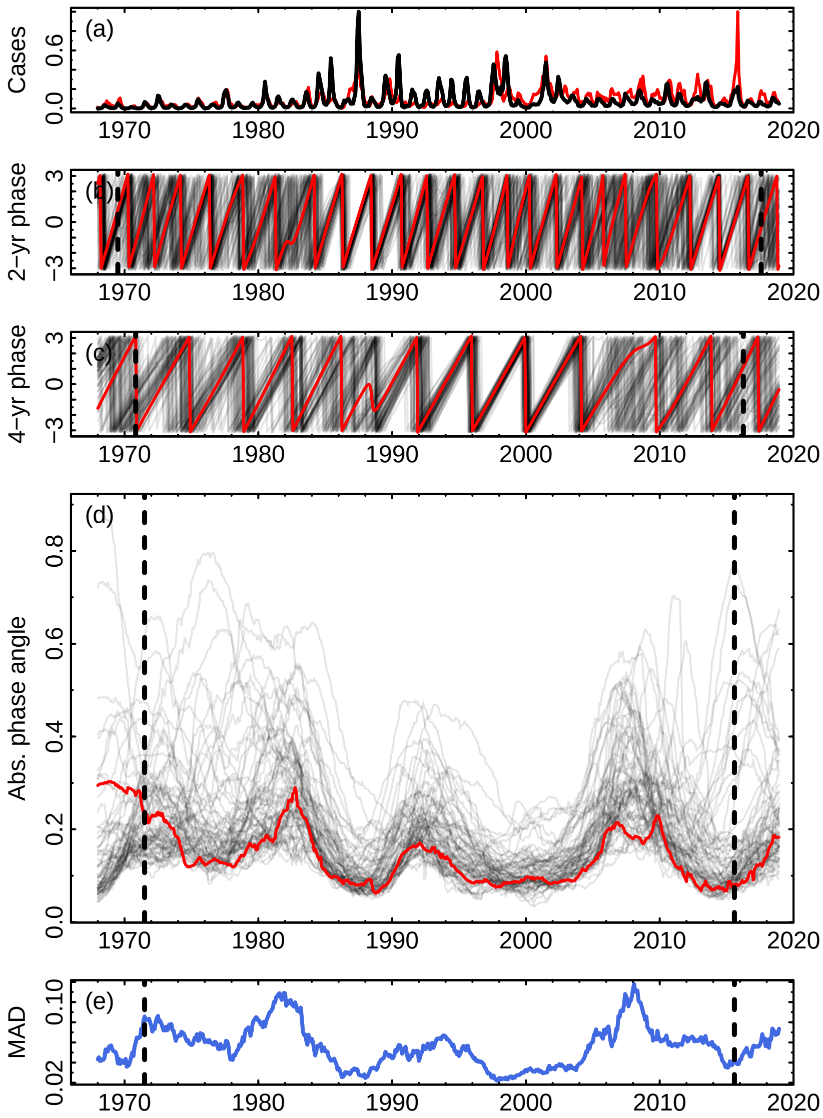

Fig S10. Synchrony using phase angles between provinces. (a) Number of cases normalised by maximum values for clarity, across the country (in black) and Bangkok (in red). (c-d) Phases at two and four year timescales. (d) Weighted absolute median phase angles per province. Thin black lines are results for each province, and red highlights Bangkok. (e) MADs of the weighted absolute median phase angles in (d), across provinces. Intervals of time during which MADs are low indicate timescales of higher synchrony. Edge effects may influence results before and after the vertical dashed lines. The underlying data are in S3 Data at `https://github.com/UF-IDD/synchrony_dengue_figures` (note (a) is the same as S8a).

Delays in peaks and nadirs

During more synchronous periods, the timing of peaks and troughs for different provinces are more likely to be similar. In this analysis we annualised the dengue counts per province (producing a number per province per year). Then, taking Bangkok as the reference time series, we took every peak and every nadir, and estimated how far in time the nearest corresponding peak and trough for every other province was. Thus, each peak and trough of the Bangkok time series had an associated distribution of distances (in time) relative to the 71 other provinces (Fig S11b). The width of these distributions indicates the similarity in the timings of peaks and nadirs across the country; the narrower the distribution, the more similar the timing, and the greater the synchrony. As an estimate of synchrony, we calculated the standard deviation for the distributions in Fig S11b (for peaks and nadirs separately); here, a low standard deviation is indicative of greater synchrony in multiannual timescales.

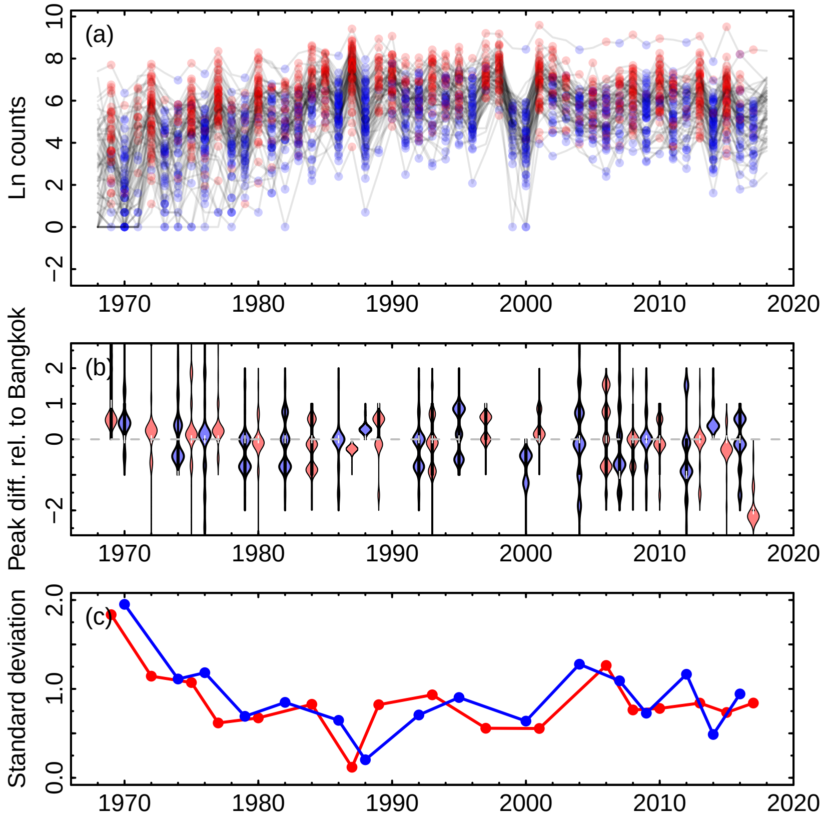

Fig S11. Synchrony comparing peaks and nadirs across provinces. (a) Annual dengue counts, where each grey line is a province. Red dots indicate maxima (peaks) in the oscillations, while blue dots show minima (nadirs). (b) Distributions of distances in years between maxima and minima of Bangkok in (a) and the corresponding nearest maxima and minima for all other provinces, centred on the timing of the maxima and minima for Bangkok. For a peak in Bangkok in 1980, if the nearest peak for Uttaradit is in 1981, then it would yield a difference of plus one year. (c) Standard deviations for the distributions of (b). Intervals of time during which standard deviations are low indicate periods of higher synchrony. The underlying data are in S3 Data at `https://github.com/UF-IDD/synchrony_dengue_figures`.

Additional simulation 1 results

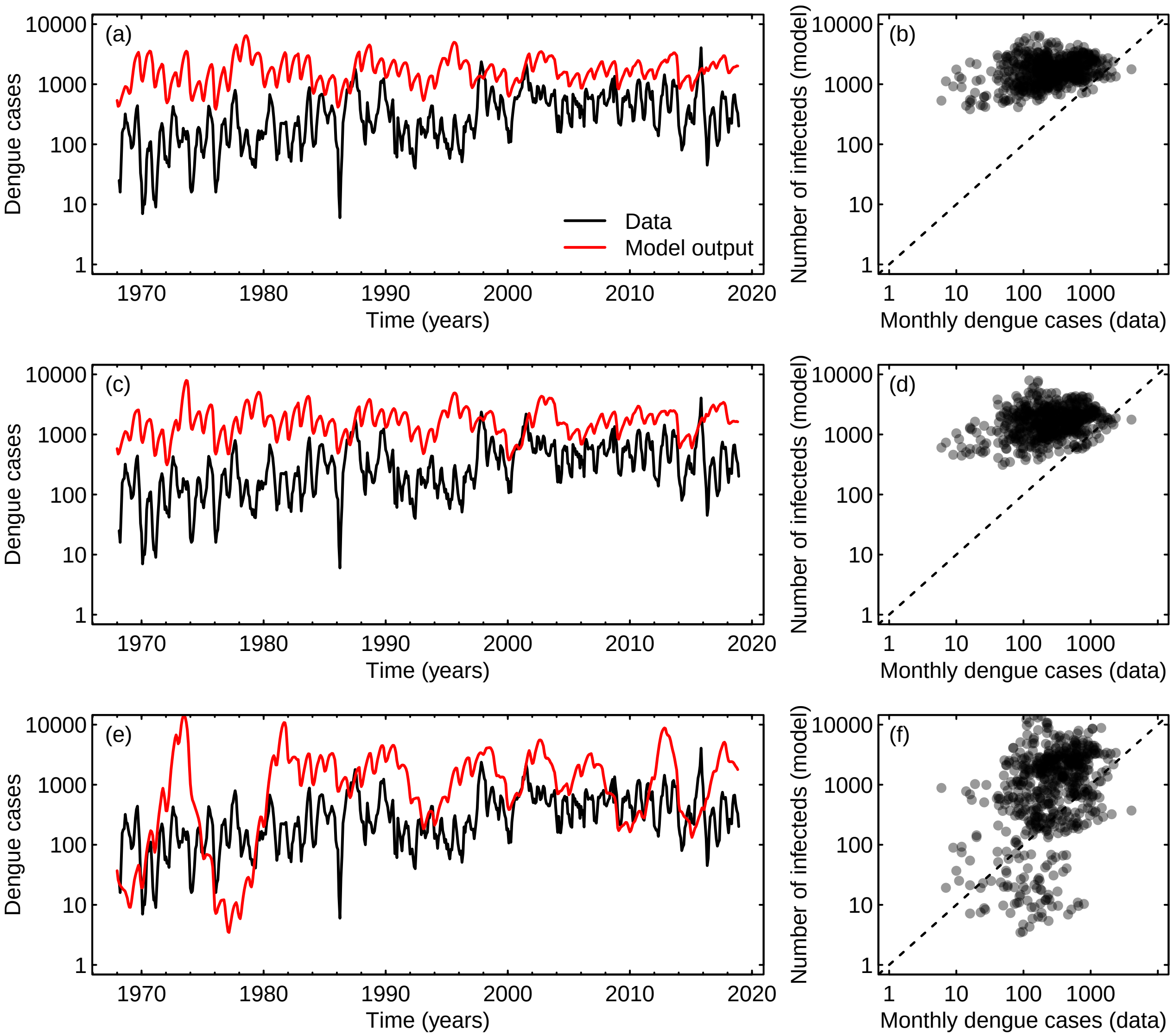

Fig S12. Comparing model output to data. (a,c,e) Example time series for the number of dengue cases in Bangkok (in black) and total number of infected individuals produced by a dengue model driven by the temperature time series in Bangkok assuming a total population of $5.6 \cdot 10^6$ (in red). (b,d,f) Direct comparison between data and model output shown in (a,c,e) respectively, the dashed line showing the $y = x$ line. Results are when using a mean cross-protection in the model of (a,b) 6 months, (c,d) one year, and (e,f) two years. The model output shows qualitative agreement with the data; quantitative differences could be ascribed to the fact that the model was here run with the same birth and death rates across all provinces. The underlying data are in S3 Data at `https://github.com/UF-IDD/synchrony_dengue_figures`.

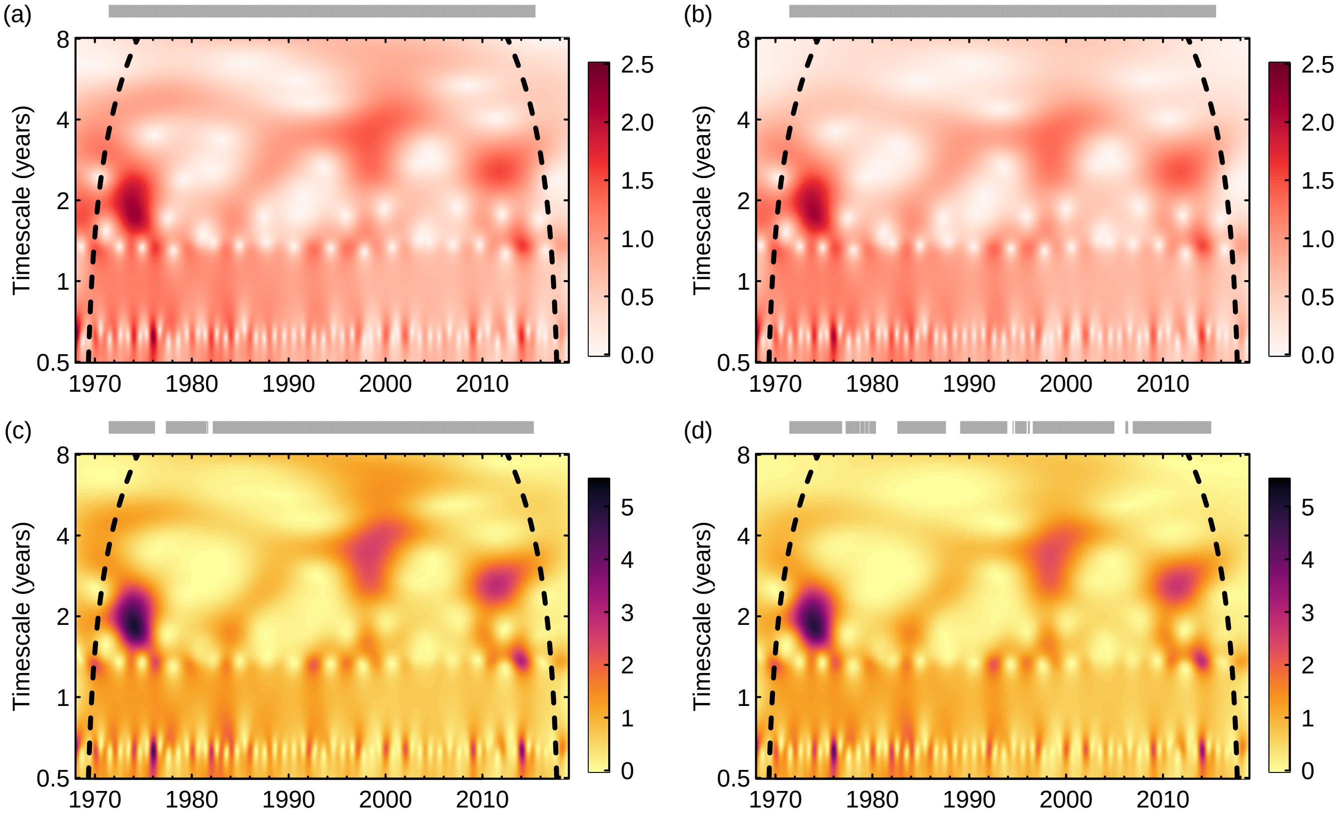

Fig S13. (Cross-)wavelet mean fields for different mean durations of cross-protection. (a,b) Wavelet mean fields for ln-transformed number of infected individuals from the model output, and (c,d) cross-wavelet mean fields between temperature (Fig 2a in the main text) and dengue (a,b), assuming a mean cross-protection of (a,c) six months and (b,d) two years. Grey bars above (c,d) indicate the times for which phases (in a,b) and phase angles (c,d) are highly consistent across locations across provinces and statistically significant (see Material and Methods in the main text); in (a,b), all points in time have statistically significant levels of synchrony. In (a,b), higher values in the mean fields indicate timescales and points in time where the phases are more consistent across provinces, and where the amplitudes of oscillations are more correlated. In (c,d), higher values correspond to timescales and points in time where the agreement between dengue and temperature is itself more consistent across provinces. Edge effects in the WTs may influence results before and after the dashed lines. The underlying data are in S3 Data at `https://github.com/UF-IDD/synchrony_dengue_figures`.

Table S1. Correlations between dengue passive surveillance data and model output WMFs. For comparison, the Pearson and Spearman correlations between dengue passive surveillance data and temperature WMFs were 0.21 (P = 0.060) and 0.20 (P = 0.069), respectively. See section “Materials and methods” in the main text for details on how the P values were estimated.

Duration of cross-protection	Pearson r (P value)	Spearman r (P value)
Six months	0.31 (0.018)	0.34 (0.011)
One year	0.29 (0.017)	0.32 (0.011)
Two years	0.28 (0.031)	0.31 (0.018)

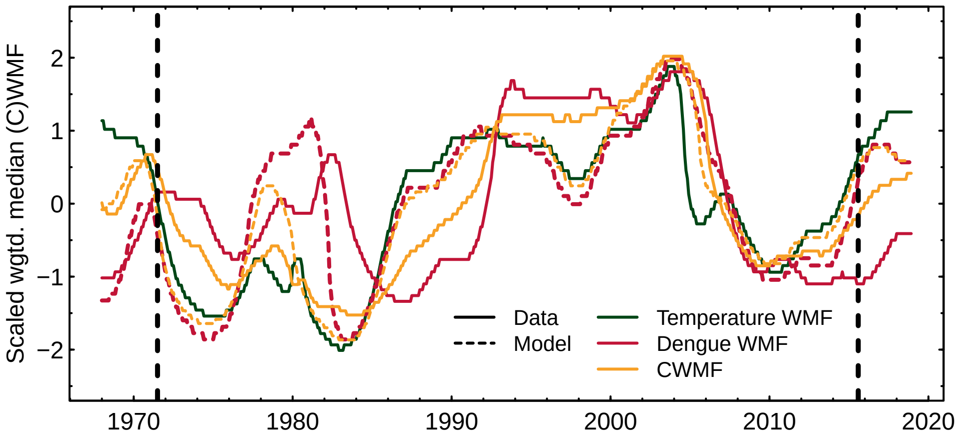

Fig S14. Weighted median timescales for (cross-)wavelet mean fields for the panels in Fig 2 in the main text. Weighted median timescales are estimated as detailed in section "Perspectives on synchrony". Edge effects in the WTs may influence results before and after the vertical dashed lines. The underlying data are in S3 Data at `https://github.com/UF-IDD/synchrony_dengue_figures`.

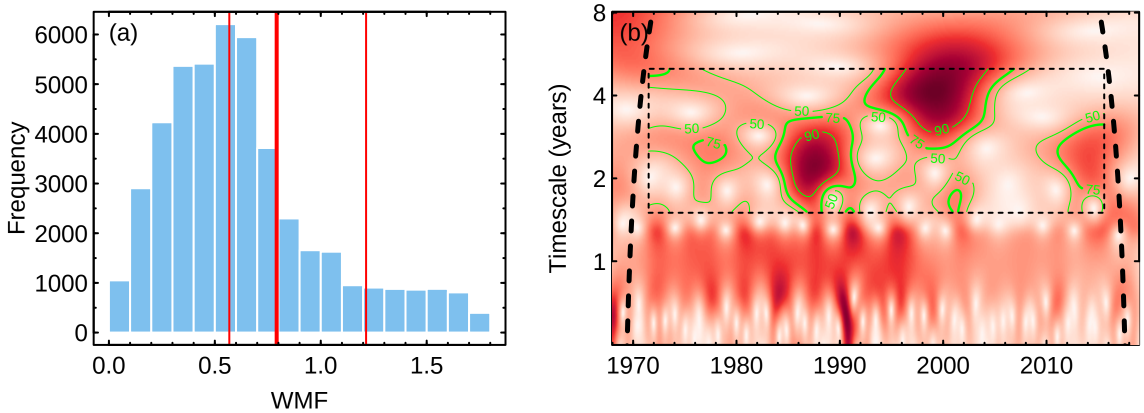

Fig S15. Defining high and low synchrony conditions. (a) Distribution of WMF values for the dengue data, focusing on multiannual timescales and time points unaffected by the cone of influence (see dashed line rectangle in (b)). The red lines show the 50th, 75th, and 90th percentiles, defining possible boundaries separating high and low synchrony. (b) WMF for dengue data, showing the contours for the three percentiles shown in (a). In the main text, we use the 75th percentile as the defining boundary. The underlying data are in S1 Data at `https://github.com/UF-IDD/synchrony_dengue_figures`.

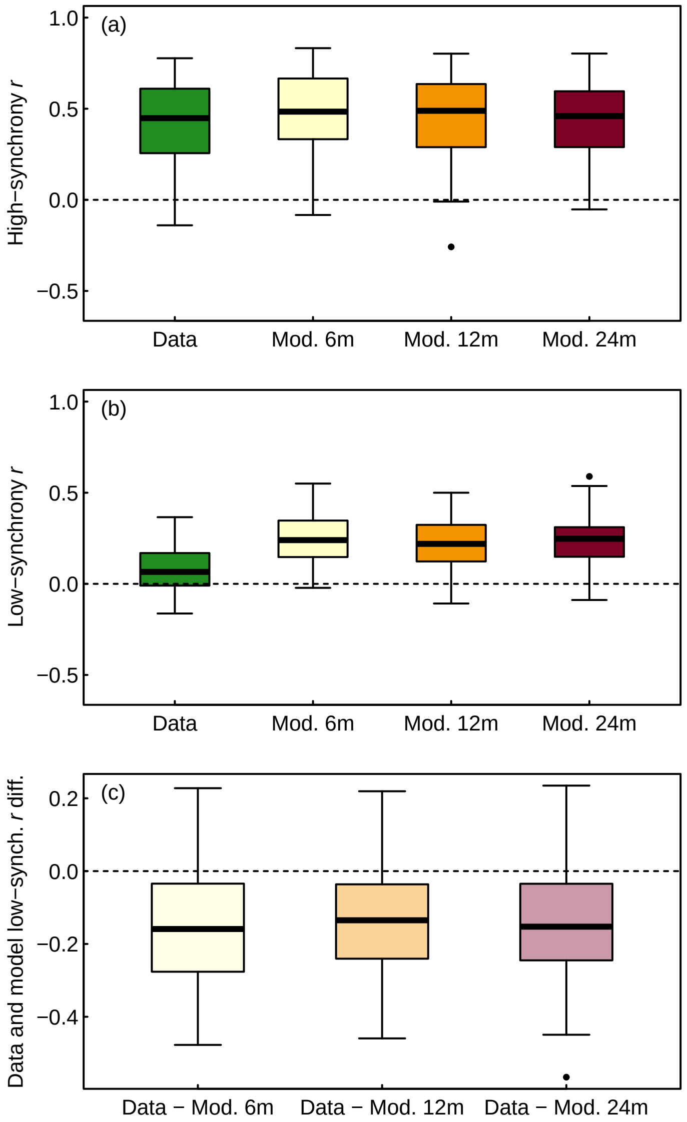

Fig S16. Distributions of correlations in wavelet power for high and low synchrony, for each province. Distributions of correlations for (a) high-synchrony conditions, (b) low-synchrony conditions, and (c) the difference between the correlations in low-synchrony conditions in the dengue data (the green boxplot in (b)) and the corresponding correlations in each of the models with different assumptions on duration of cross-protection (the other boxplots in (b)). Here we use the 75th percentile as the boundary separating high and low-synchrony conditions. "Data" shows distributions of correlations (one correlation value per province) in wavelet power between the dengue data and temperature wavelet spectra. The model ("Mod.") boxplots show the distributions of correlations in wavelet power between the dengue model output and dengue data, for three different assumptions on the duration of mean cross-protection. A negative value in (c) means that in a province, the low-synchrony correlation between dengue data and temperature was lower than between dengue model output and dengue data. High-synchrony correlations are high in both data and model, but low-synchrony correlations are lower in data than in the model comparisons. See Fig S15 for how high and low-synchrony conditions are defined, and see section "Materials and methods" in the main text for further details on methods used, and Table S2 for summary values. Alternative results using different percentiles as the boundaries between high and low-synchrony conditions are shown in Figs S17 and S18. The underlying data are in S3 Data at `https://github.com/UF-IDD/synchrony_dengue_figures`.

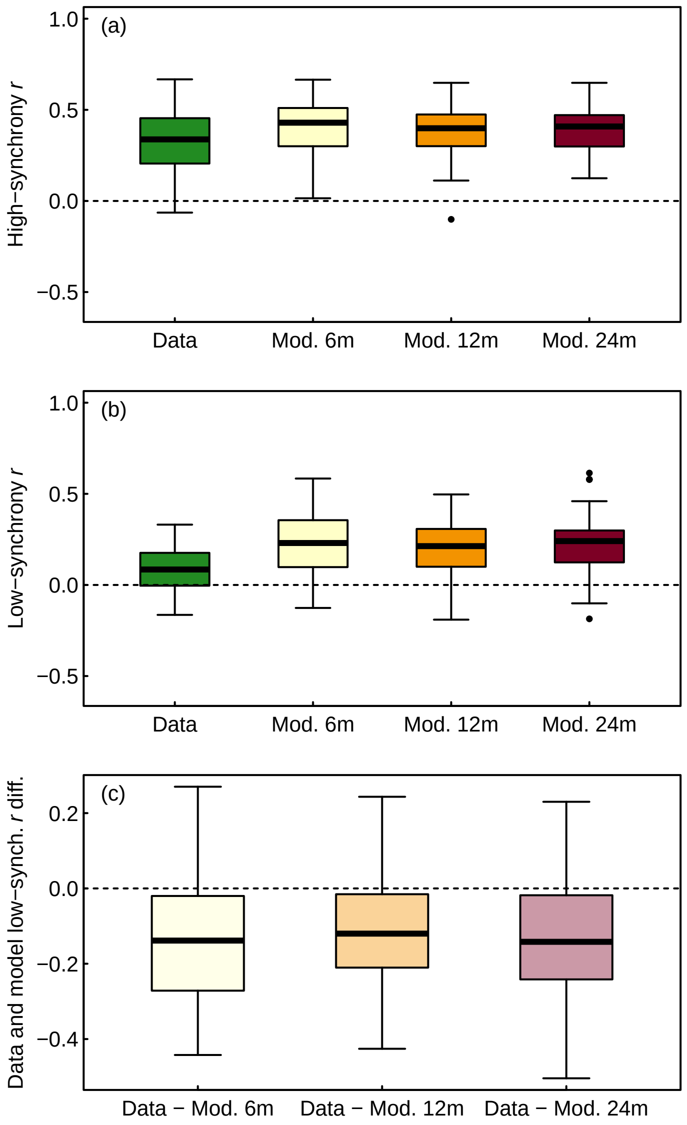

Fig S17. Distributions of correlations in wavelet power for high and low synchrony, for alternative definitions of high synchrony. Alternative results to those shown in Fig S16, but using the 50th percentile as the boundary separating high and low-synchrony conditions. See Fig S15 for how high and low-synchrony conditions are defined, and see section Materials and methods in the main text for further details on methods used, and Table S2 for summary values. The underlying data are in S3 Data at `https://github.com/UF-IDD/synchrony_dengue_figures`.

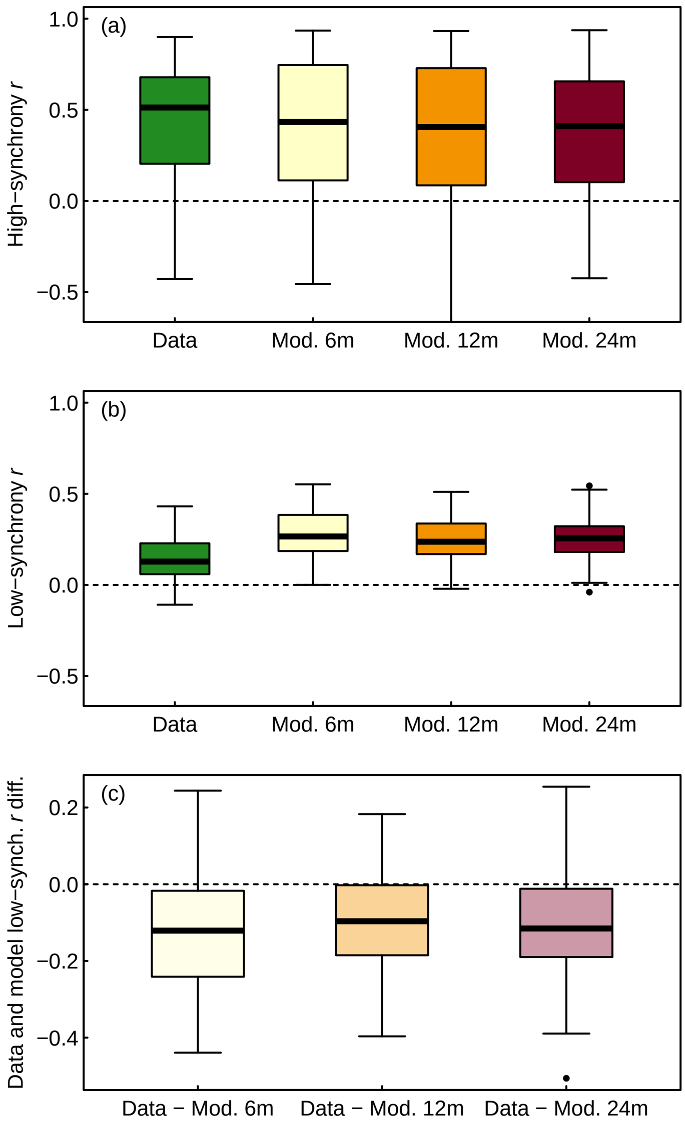

Fig S18. Distributions of correlations in wavelet power for high and low synchrony, for alternative definitions of high synchrony. Alternative results to those shown in Fig S16, but using the 90th percentile as the boundary separating high and low-synchrony conditions. See Fig S15 for how high and low-synchrony conditions are defined, and see section Materials and methods in the main text for further details on methods used, and Table S2 for summary values. The underlying data are in S3 Data at `https://github.com/UF-IDD/synchrony_dengue_figures`.

Table S2. Correlations during high and low synchrony. Summaries of mean Spearman correlations between the wavelet spectra of temperature and dengue data (for "Data"), and between dengue data and modelled dengue output (for "Model"), separately for high and low-synchrony conditions, across all provinces. "Percentile" is the percentile of the WMF values that defines the boundary between high and low-synchrony conditions (Fig S15); in the main text we use the 75th percentile as the boundary. "Difference" gives the mean difference in the Spearman correlations between high and low-synchrony conditions. P-values are for a two-tailed t-test. See section "Materials and methods" in the main text for details on the methods.

Percentile	*Comparison*	*Synchrony*	*Mean Spearman r*	*P-value*
75	Data	High	0.42	$< 2.2 \cdot 10^{-16}$
		Low	0.09	$1.6 \cdot 10^{-7}$
		Difference	0.33	$< 2.2 \cdot 10^{-16}$
	Model (6m cross-protection)	High	0.47	$< 2.2 \cdot 10^{-16}$
		Low	0.24	$< 2.2 \cdot 10^{-16}$
		Difference	0.23	$3.6 \cdot 10^{-11}$
	Model (12m cross-protection)	High	0.44	$< 2.2 \cdot 10^{-16}$
		Low	0.22	$< 2.2 \cdot 10^{-16}$
		Difference	0.22	$1.6 \cdot 10^{-10}$
	Model (24m cross-protection)	High	0.44	$< 2.2 \cdot 10^{-16}$
		Low	0.24	$< 2.2 \cdot 10^{-16}$
		Difference	0.20	$2.3 \cdot 10^{-10}$
50	Data	High	0.34	$< 2.2 \cdot 10^{-16}$
		Low	0.09	$1.4 \cdot 10^{-7}$
		Difference	0.25	$< 2.2 \cdot 10^{-16}$
	Model (6m cross-protection)	High	0.41	$< 2.2 \cdot 10^{-16}$
		Low	0.22	$< 2.2 \cdot 10^{-16}$
		Difference	0.19	$4.4 \cdot 10^{-13}$
	Model (12m cross-protection)	High	0.38	$< 2.2 \cdot 10^{-16}$
		Low	0.21	$< 2.2 \cdot 10^{-16}$
		Difference	0.18	$3.0 \cdot 10^{-13}$
	Model (24m cross-protection)	High	0.40	$< 2.2 \cdot 10^{-16}$
		Low	0.22	$< 2.2 \cdot 10^{-16}$
		Difference	0.17	$9.8 \cdot 10^{-14}$
90	Data	High	0.42	$8.2 \cdot 10^{-16}$
		Low	0.15	$4.5 \cdot 10^{-16}$
		Difference	0.27	$2.6 \cdot 10^{-10}$
	Model (6m cross-protection)	High	0.40	$1.5 \cdot 10^{-13}$
		Low	0.27	$< 2.2 \cdot 10^{-16}$
		Difference	0.13	$2.2 \cdot 10^{-3}$
	Model (12m cross-protection)	High	0.36	$1.3 \cdot 10^{-11}$
		Low	0.25	$< 2.2 \cdot 10^{-16}$
		Difference	0.12	$9.4 \cdot 10^{-3}$
	Model (24m cross-protection)	High	0.36	$3.4 \cdot 10^{-12}$
		Low	0.26	$< 2.2 \cdot 10^{-16}$
		Difference	0.11	$1.1 \cdot 10^{-2}$

Additional simulations 2 and 3 results

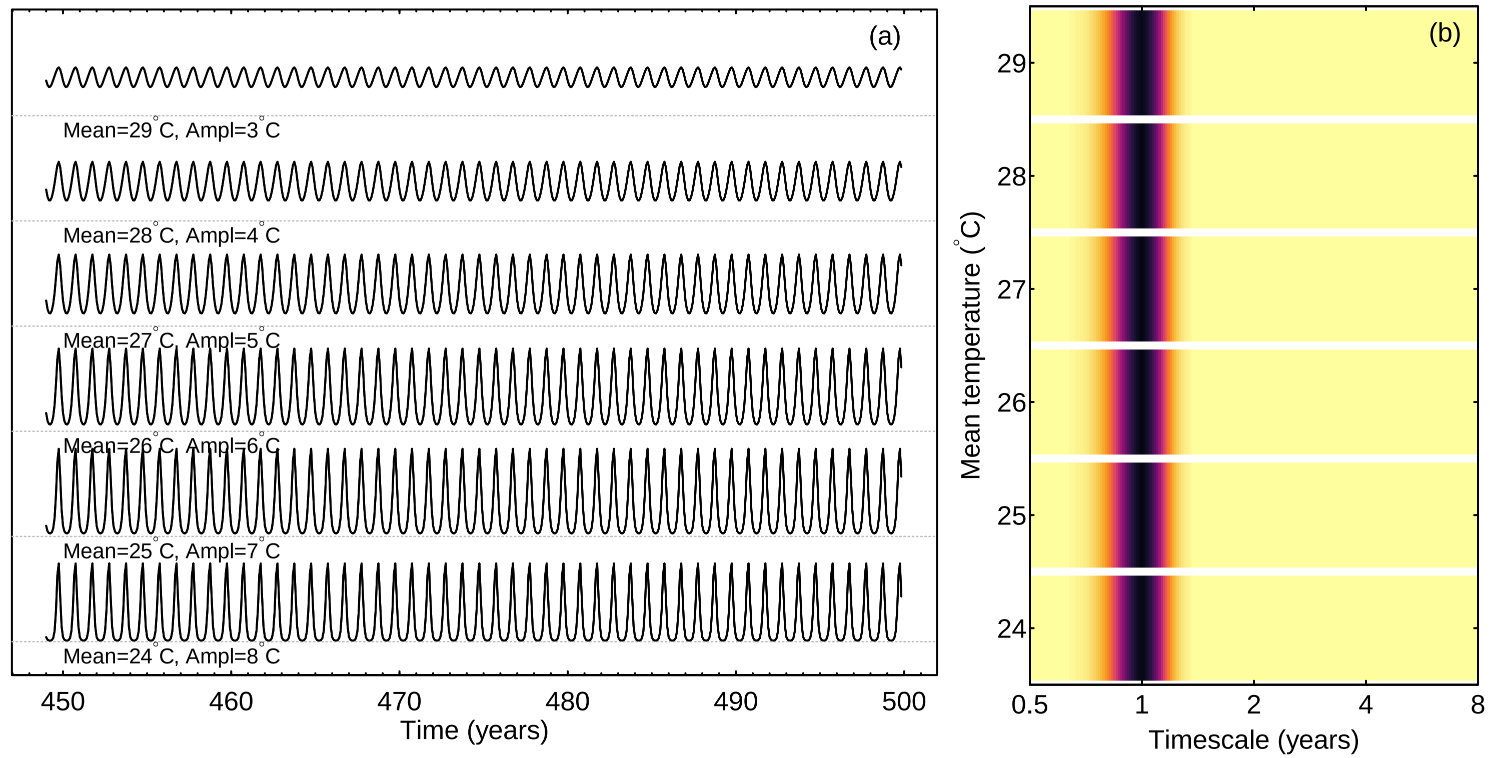

Fig S19. Effect of temperature on multiannual dynamics, assuming a mean cross-protection of six months. In both panels, each row corresponds to a hypothetical location experiencing a different temperature regime, going from low to high mean temperature from bottom to top (see panel a). (a) Time series of total number of infected individuals (on linear scale) for the last 51 years of each simulation. The time series are plotted to scale. (b) Average wavelet power per timescale, where darker colours mean larger power. Here, the only multiannual periodicities detected were of very low power with period > 14 years, and are therefore not shown. The underlying data are in S3 Data at `https://github.com/UF-IDD/synchrony_dengue_figures`.

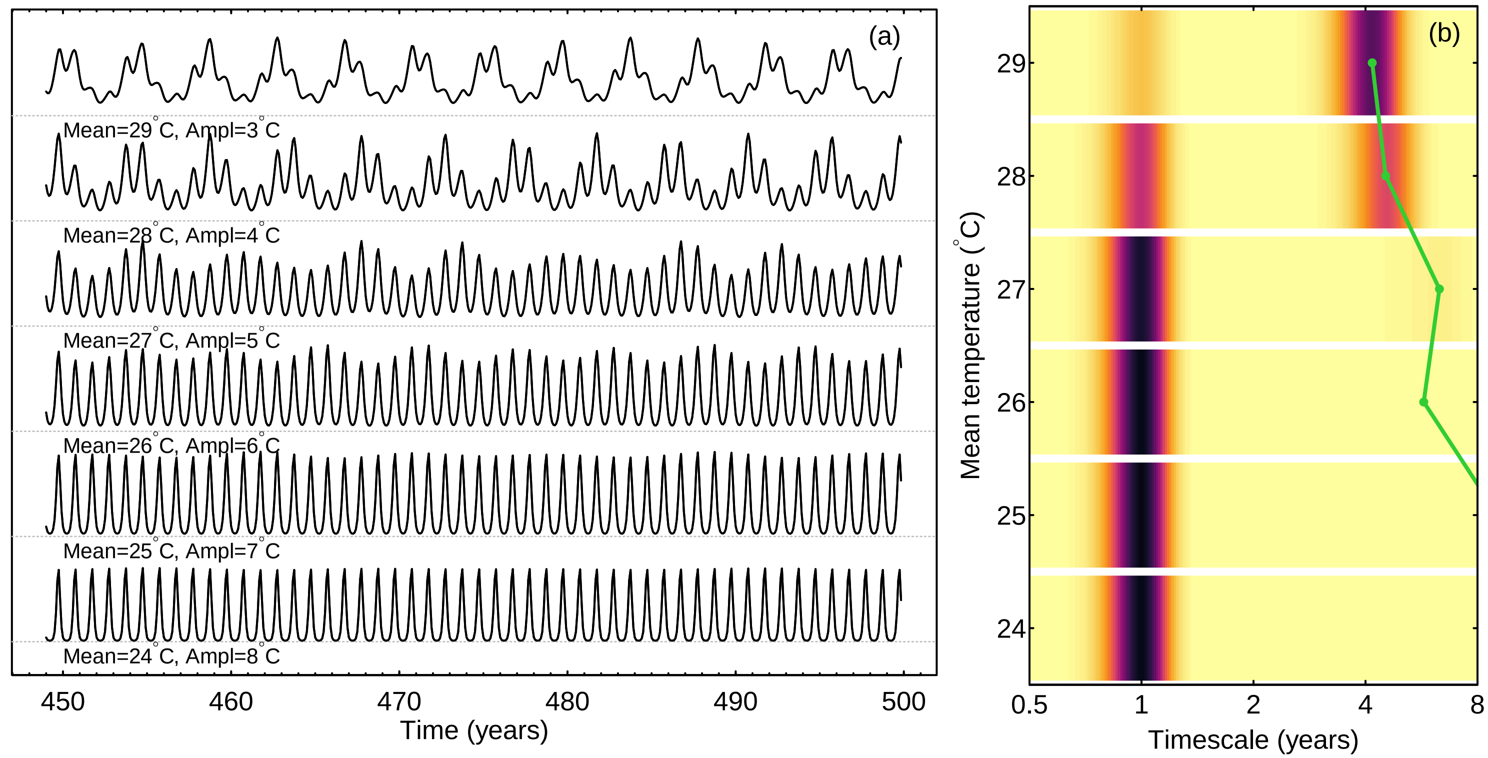

Fig S20. Effect of temperature on multiannual dynamics, assuming a mean cross-protection of one year. In both panels, each row corresponds to a hypothetical location experiencing a different temperature regime, going from low to high mean temperature from bottom to top (see panel a). (a) Time series of total number of infected individuals (on linear scale) for the last 51 years of each simulation. The time series are plotted to scale. (b) Average wavelet power per timescale, where darker colours mean larger power, and green lines show multiannual peaks. The underlying data are in S3 Data at `https://github.com/UF-IDD/synchrony_dengue_figures`.

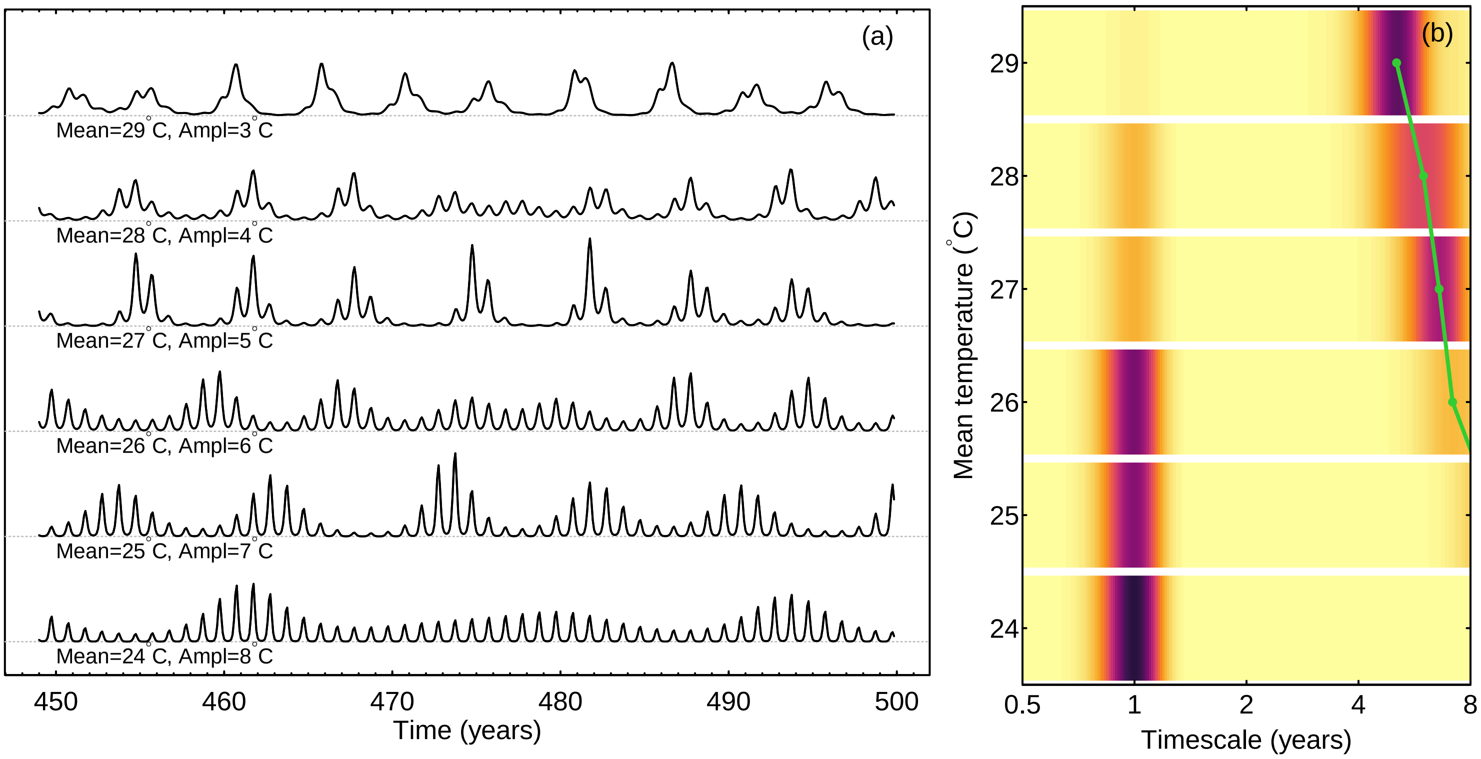

Fig S21. Effect of temperature on multiannual dynamics, assuming a mean cross-protection of two years. In both panels, each row corresponds to a hypothetical location experiencing a different temperature regime, going from low to high mean temperature from bottom to top (see panel a). (a) Time series of total number of infected individuals (on linear scale) for the last 51 years of each simulation. The time series are plotted to scale. (b) Average wavelet power per timescale, where darker colours mean larger power, and green lines show multiannual peaks. The underlying data are in S3 Data at `https://github.com/UF-IDD/synchrony_dengue_figures`.

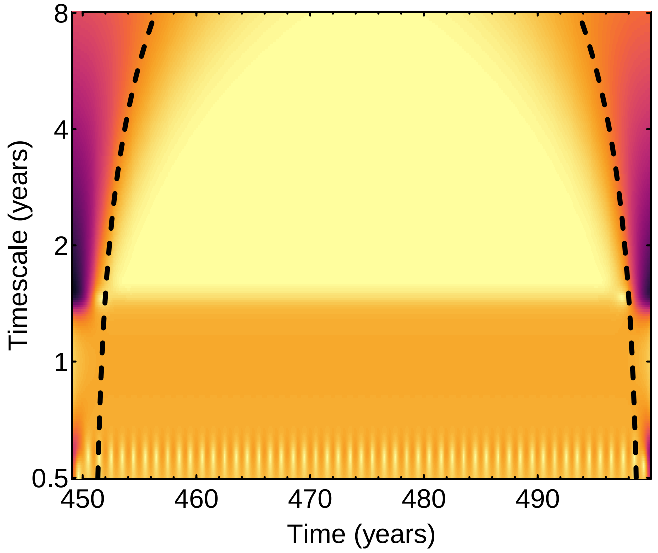

Fig S22. WMF for dengue dynamics across six hypothetical locations with different thermal regimes, assuming a mean cross-protection of 6 months (see Fig S19). Here, temperature is assumed to be sinusoidal (seasonal cycles). Darker colours indicate greater consistency (synchrony) across locations. Edge effects in the WTs may influence results before and after the dashed lines. The underlying data are in S3 Data at `https://github.com/UF-IDD/synchrony_dengue_figures`.

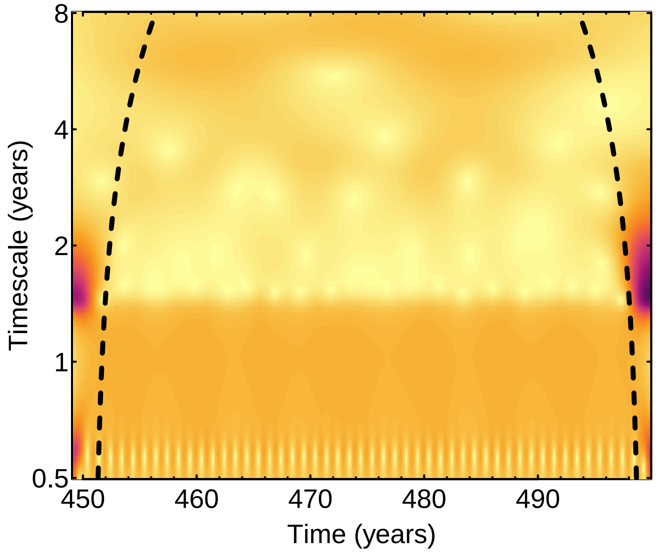

Fig S23. WMF for dengue dynamics across six hypothetical locations with different thermal regimes, assuming a mean cross-protection of two years (see Fig S21). Here, temperature is assumed to be sinusoidal (seasonal cycles). Darker colours indicate greater consistency (synchrony) across locations. Edge effects in the WTs may influence results before and after the dashed lines. The underlying data are in S3 Data at `https://github.com/UF-IDD/synchrony_dengue_figures`.

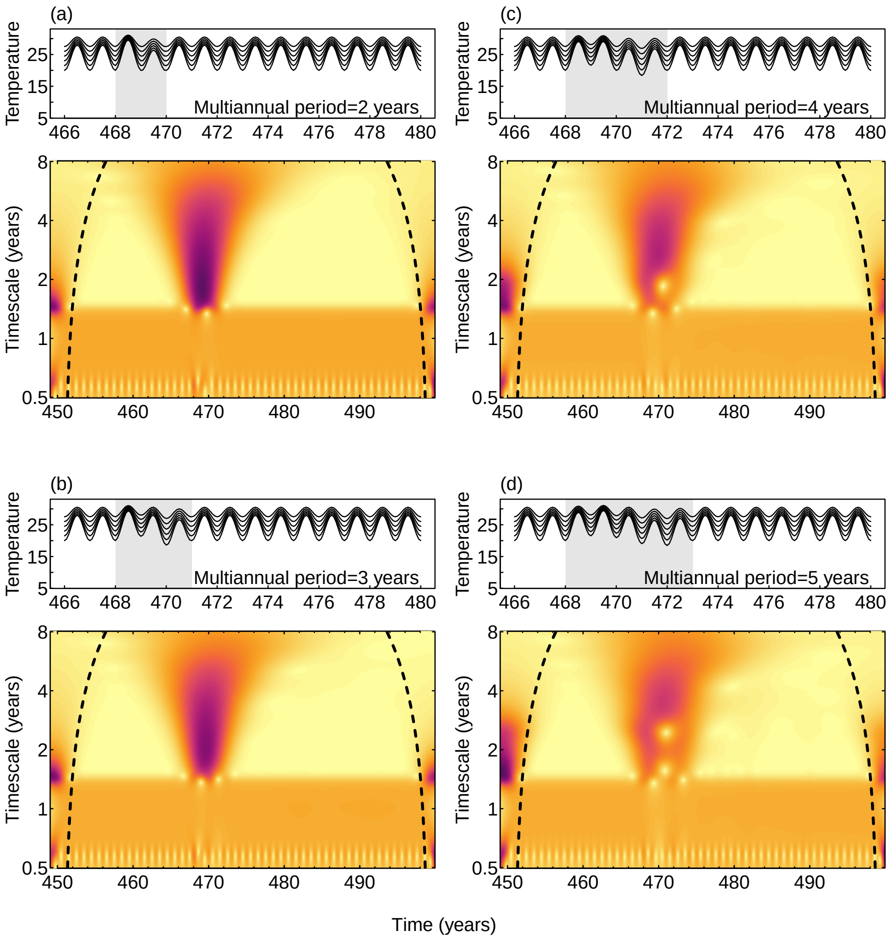

Fig S24. Single multiannual fluctuations. WMFs for dengue dynamics across six hypothetical locations with different thermal regimes, assuming a mean cross-protection of six months. Here, temperature is assumed to be sinusoidal, with the exception of a single multiannual fluctuation (highlighted using a grey box), with a different period in each panel (a–d). The multiannual fluctuation has an amplitude 0.4 times that of the seasonal cycle. The time series in each panel show a snippet of the temperatures across the six hypothetical locations, while the heat maps show the WMF in each case, where darker colours indicate greater consistency (synchrony) across locations. Fig S22 shows the WMF without multiannual fluctuations. Simulations were repeated starting the multiannual fluctuation at four different times of year and on ten different years, with the same result. Edge effects in the WTs may influence results before and after the dashed lines. The underlying data are in S4 Data at `https://github.com/UF-IDD/synchrony_dengue_figures`.

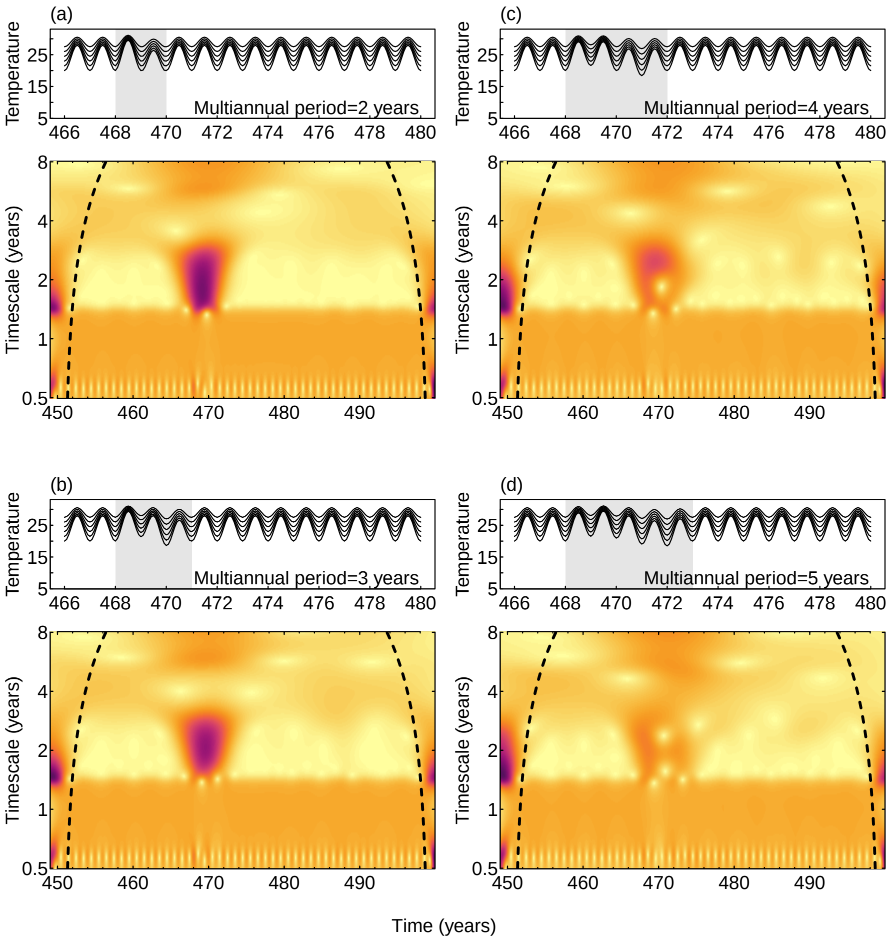

Fig S25. Single multiannual fluctuation. Here, we assume a mean cross-protection of one year. The multiannual fluctuation has an amplitude 0.4 times that of the seasonal cycle. Fig 4d in the main text shows the WMF when there is no multiannual fluctuation. See caption to Fig S24 for details. The underlying data are in S4 Data at `https://github.com/UF-IDD/synchrony_dengue_figures`.

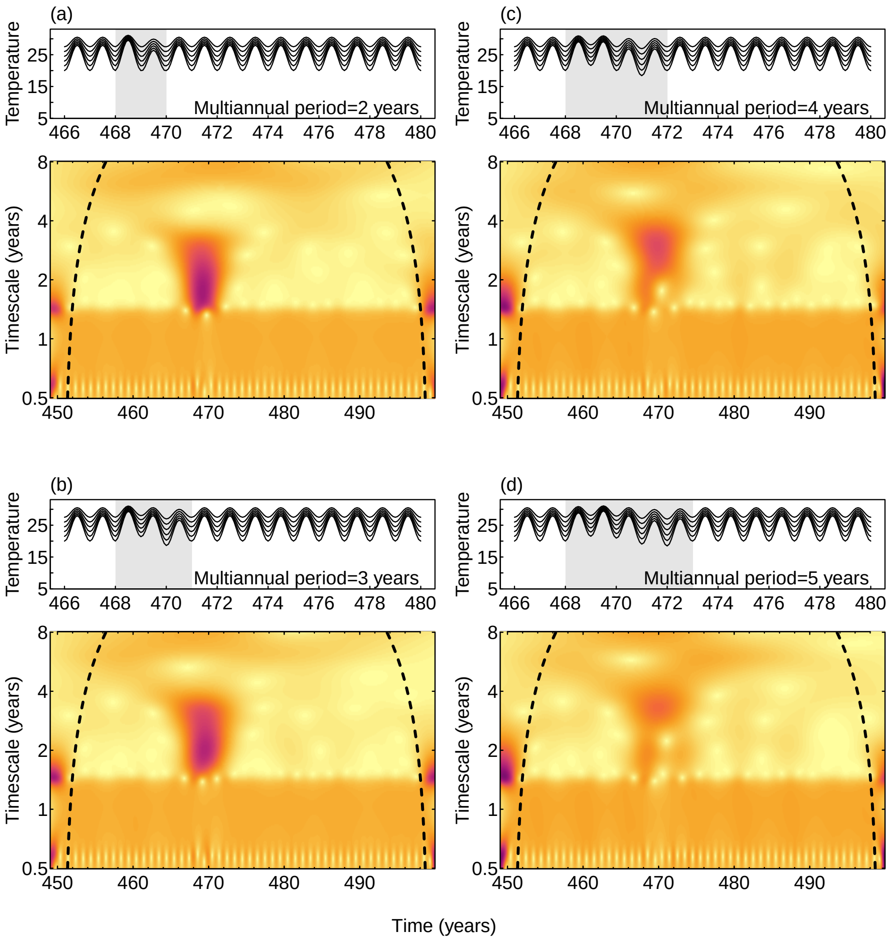

Fig S26. Single multiannual fluctuation. Here, we assume a mean cross-protection of two years. The multiannual fluctuation has an amplitude 0.4 times that of the seasonal cycle. Fig S23 shows the WMF when there is no multiannual fluctuation. See caption to Fig S24 for details. The underlying data are in S4 Data at `https://github.com/UF-IDD/synchrony_dengue_figures`.

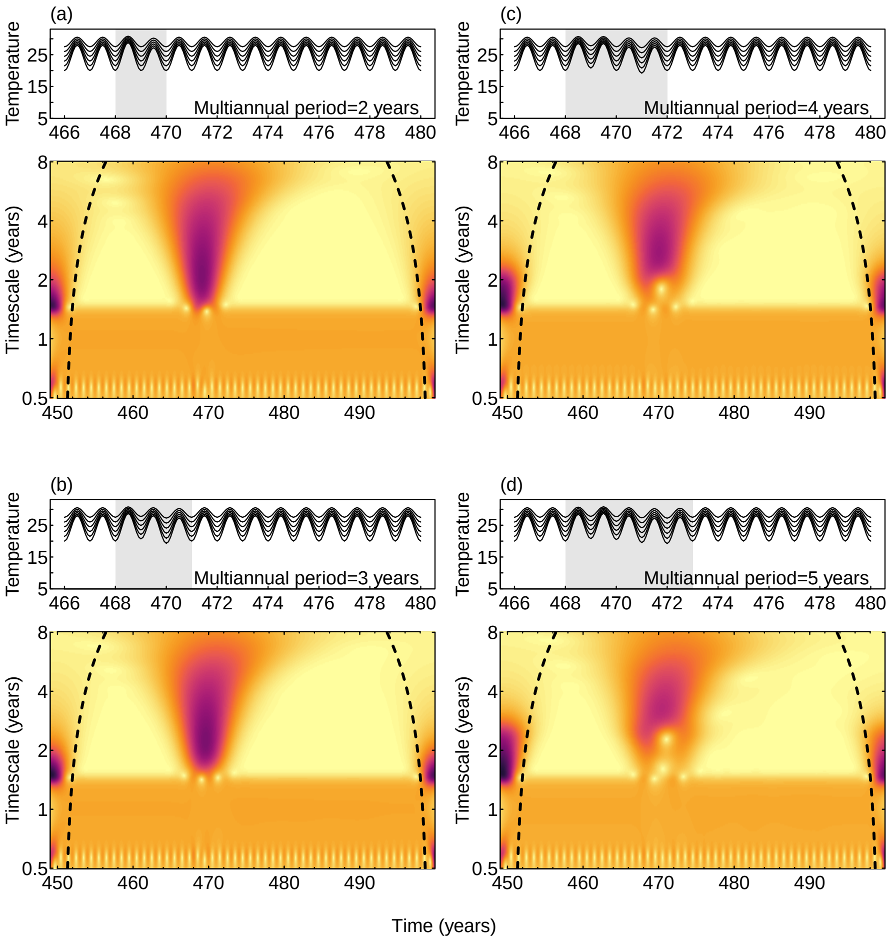

Fig S27. Single multiannual fluctuation. Here, we assume a mean cross-protection of one year. The multiannual fluctuation has an amplitude 0.2 times that of the seasonal cycle. Fig S22 shows the WMF when there is no multiannual fluctuation. See caption to Fig S24 for details. The underlying data are in S5 Data at `https://github.com/UF-IDD/synchrony_dengue_figures`.

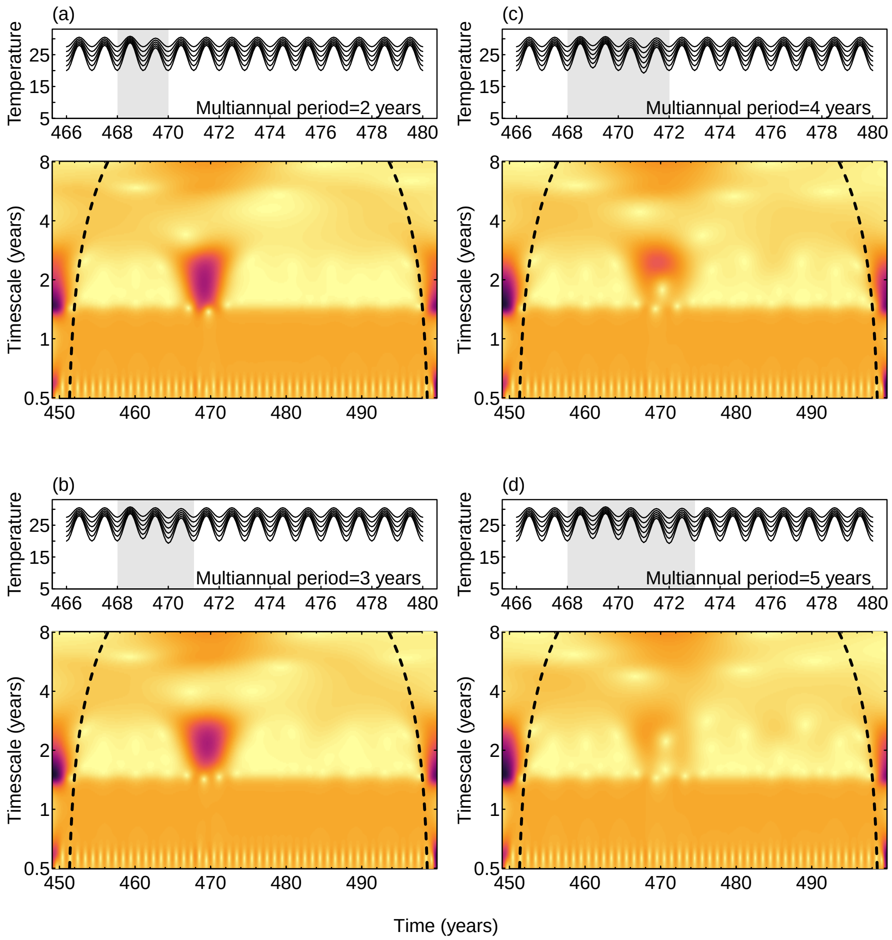

Fig S28. Single multiannual fluctuation. Here, we assume a mean cross-protection of one year. The multiannual fluctuation has an amplitude 0.2 times that of the seasonal cycle. Fig 4d in the main text shows the WMF when there is no multiannual fluctuation. See caption to Fig S24 for details. The underlying data are in S5 Data at `https://github.com/UF-IDD/synchrony_dengue_figures`.

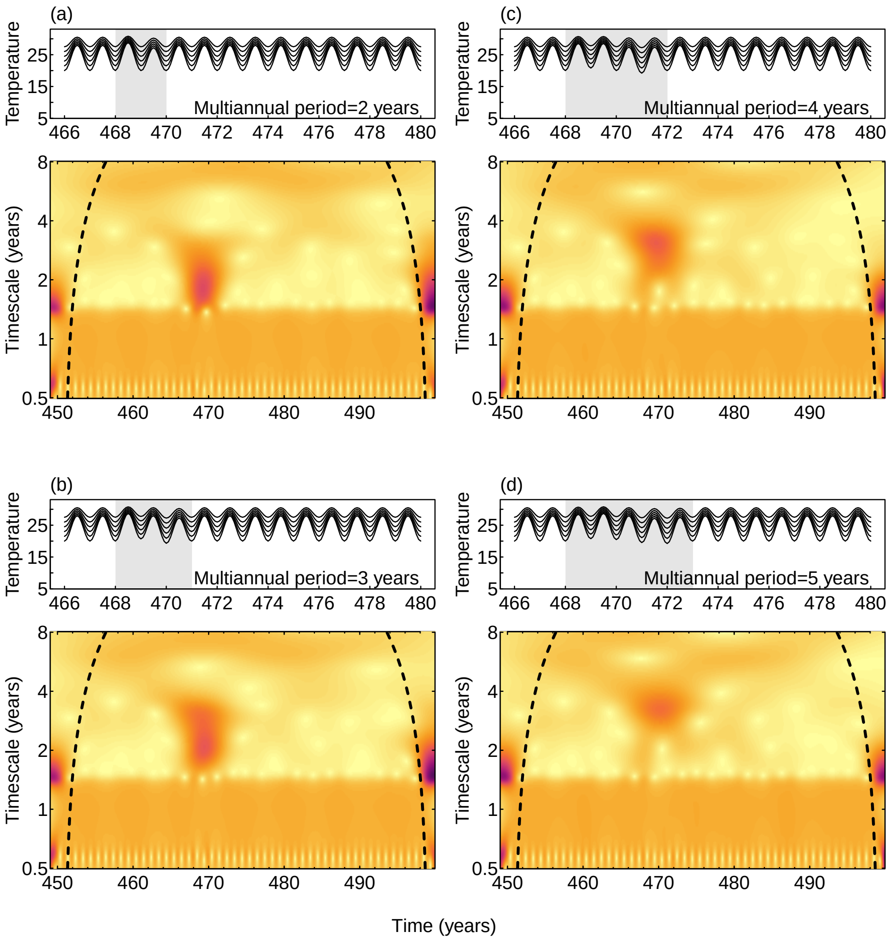

Fig S29. Single multiannual fluctuation. Here, we assume a mean cross-protection of two years. The multiannual fluctuation has an amplitude 0.2 times that of the seasonal cycle. Fig S23 shows the WMF when there is no multiannual fluctuation. See caption to Fig S24 for details. The underlying data are in S5 Data at `https://github.com/UF-IDD/synchrony_dengue_figures`.

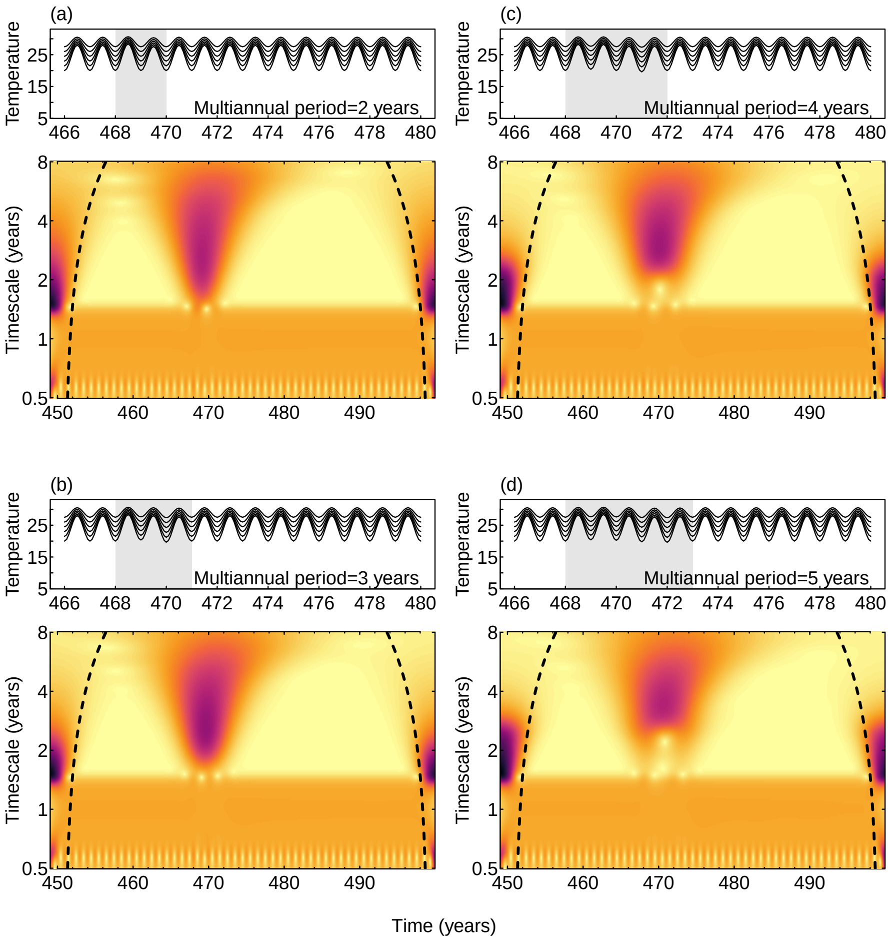

Fig S30. Single multiannual fluctuation. Here, we assume a mean cross-protection of six months. The multiannual fluctuation has an amplitude 0.1 times that of the seasonal cycle. Fig S22 shows the WMF when there is no multiannual fluctuation. See caption to Fig S24 for details. The underlying data are in S6 Data at `https://github.com/UF-IDD/synchrony_dengue_figures`.

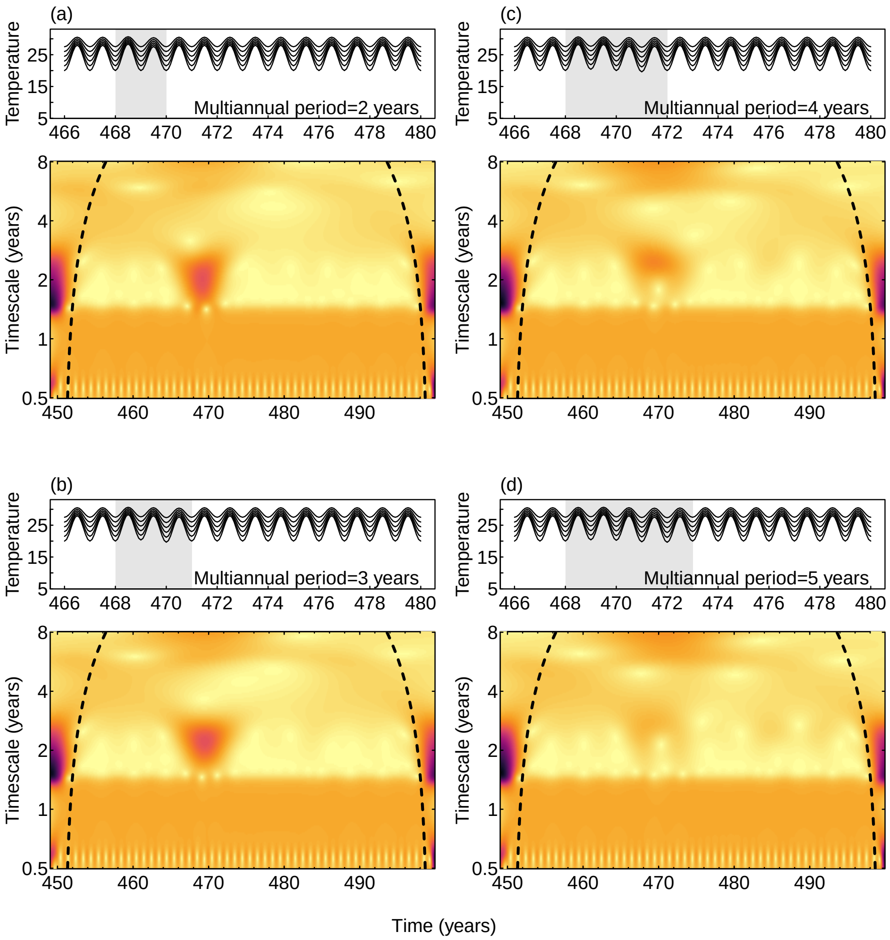

Fig S31. Single multiannual fluctuation. Here, we assume a mean cross-protection of one year. The multiannual fluctuation has an amplitude 0.1 times that of the seasonal cycle. Fig 4d in the main text shows the WMF when there is no multiannual fluctuation. See caption to Fig S24 for details. The underlying data are in S6 Data at `https://github.com/UF-IDD/synchrony_dengue_figures`.

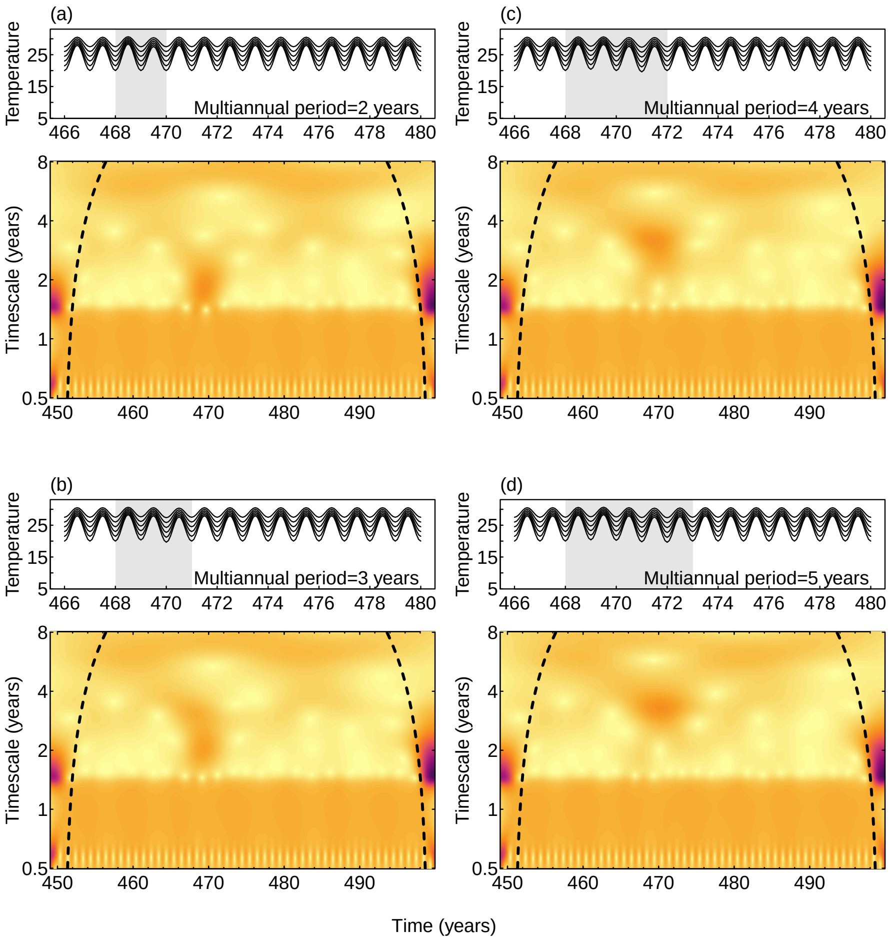

Fig S32. Single multiannual fluctuation. Here, we assume a mean cross-protection of two years. The multiannual fluctuation has an amplitude 0.1 times that of the seasonal cycle. Fig S23 shows the WMF when there is no multiannual fluctuation. See caption to Fig S24 for details. The underlying data are in S6 Data at `https://github.com/UF-IDD/synchrony_dengue_figures`.

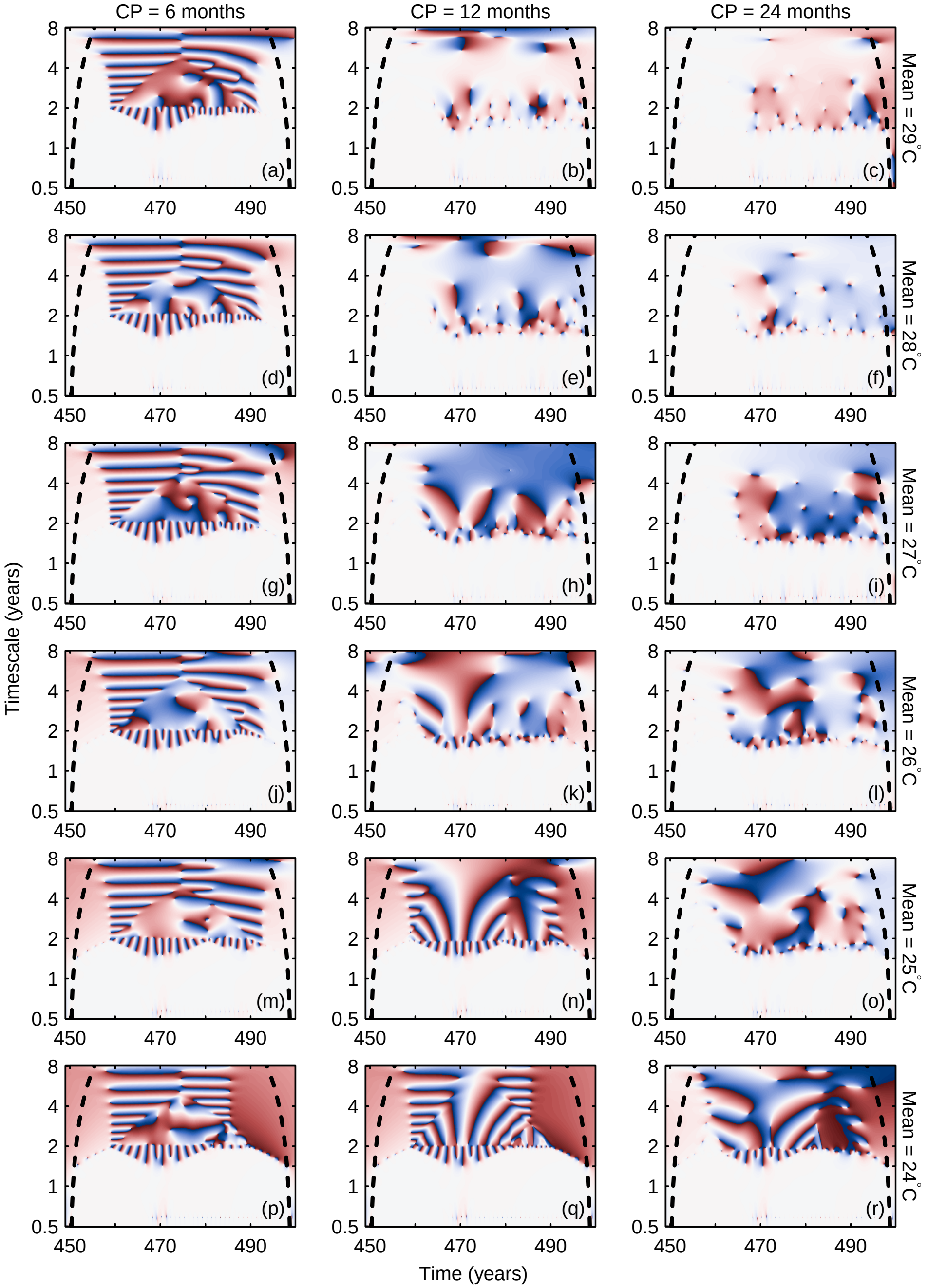

Fig S33. Phase angles between simulations with and without a a single multiannual fluctuation (with a timescale of four years) in temperature. Panels show the phase angle between simulations with and without a single multiannual (four-year) fluctuation in temperature superimposed on the seasonal cycle. Here, the amplitude of the multiannual fluctuation is 0.2 times that of the seasonal cycle. Colors range from dark red (a phase angle of $-\pi$) to dark blue (a phase angle of π), passing through white (with a phase angle of zero; i.e., both simulations are in phase). The multiannual fluctuation is introduced on year 468. Columns correspond to different mean durations of cross-protection, and rows are temperature regimes with a different mean temperature. Note how the top-right panel shows there is little difference in the dynamics of dengue with and without a multiannual fluctuation in temperature (i.e., dengue is insensitive to the multiannual fluctuation), but as mean temperatures and/or cross-protections are reduced, the differences between the two simulations becomes evident. Impacts of the single multiannual fluctuation can also be long-lasting. Results for different amplitudes and timescales of the multiannual fluctuation are qualitatively similar. Edge effects in the WTs may influence results before and after the dashed lines. The underlying data are in S7 Data to S12 Data at `https://github.com/UF-IDD/synchrony_dengue_figures`.

Additional results on synchrony in temperature

Here we show similar results as those shown in previous sections, but for temperature. We focus on weighted median timescales and moving window spline correlograms.

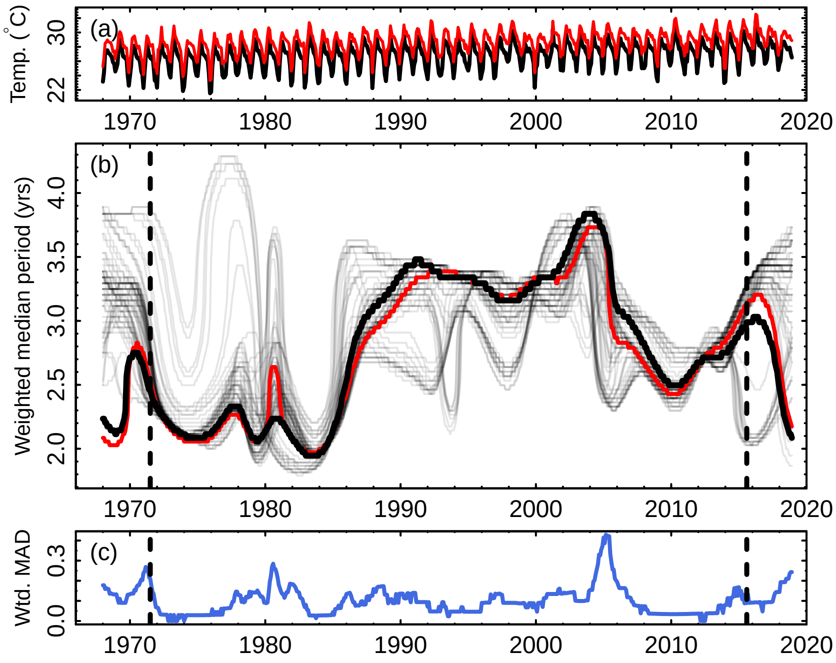

Fig S34. Spatial synchrony in temperature using weighted median timescales. (a) Mean temperature across Thailand, and temperature for Bangkok. (b) Weighted median timescales, indicating the dominant multiannual timescale for each province at each point in time (see section "Perspectives on synchrony"). (c) The median absolute deviation (MADs), estimated on the time series shown in panel (b), which measures the relative dispersion of the weighted median timescales across provinces at each point in time, while accounting for their uncertainty. Each gray line corresponds to a province, Bangkok is highlighted in red, and black corresponds to the estimate for the total counts across all provinces. Periods during which the weighted MADs in (c) are lower correspond to higher degrees of synchrony. Edge effects in the WTs may influence results before and after the vertical dashed lines. The underlying data are in S13 Data at `https://github.com/UF-IDD/synchrony_dengue_figures`.

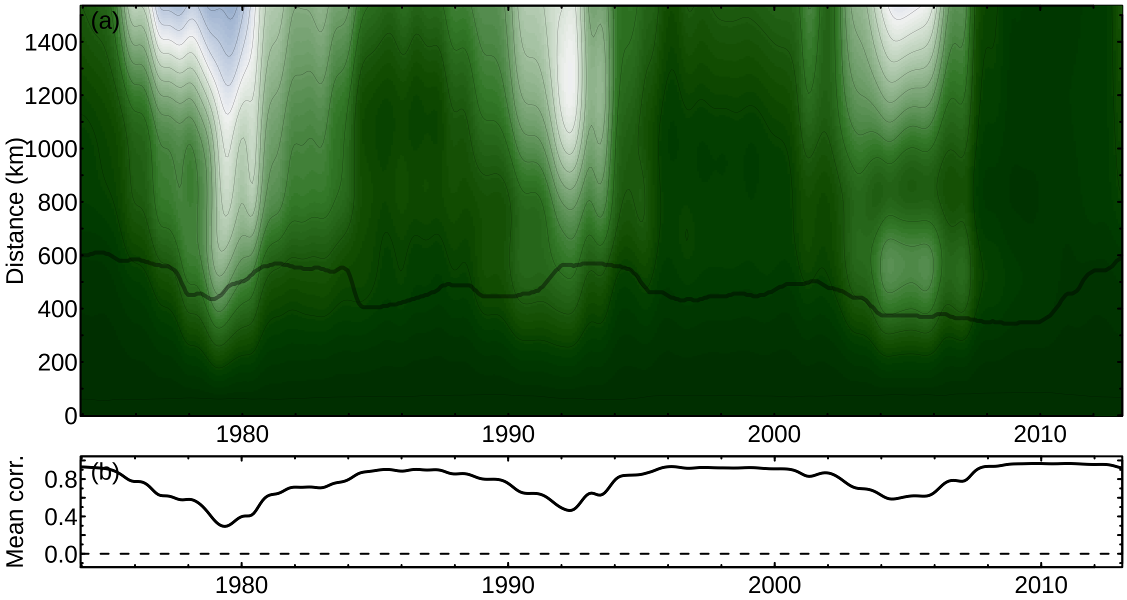

Fig S35. Synchrony in temperature using spline correlograms. (a) Spline correlograms for a five-year moving window (with time on the x-axis and pairwise distances between Thai provinces on the y-axis). The colour indicates the pairwise correlation in reconstructions of time series using multiannual components only. Green, white, and blue are positive, zero, and negative correlations, respectively. The thick grey line shows the distance at each point in time for which the pairwise correlation is no greater than could be expected at random between any two provinces. (b) Average correlation across all distances within the five-year window, for each point in time. The underlying data are in S13 Data at `https://github.com/UF-IDD/synchrony_dengue_figures`.

Patterns in temperature across Thailand

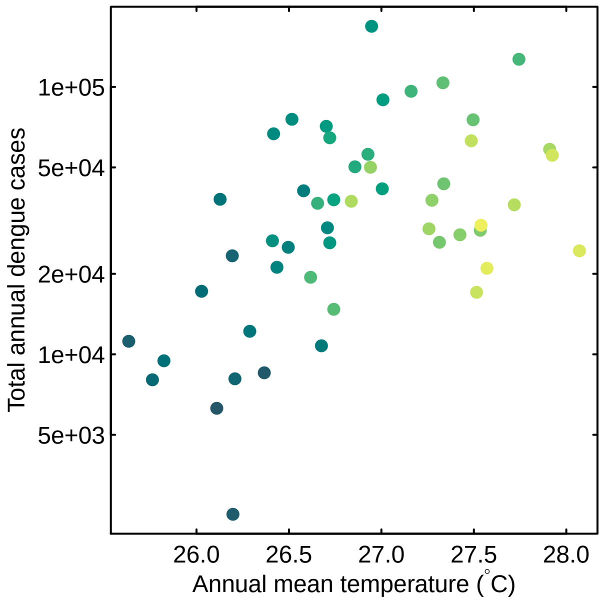

Fig S36. Ln total number of annual dengue cases across Thailand as a function of the mean annual temperature for 1968–2018, where each point corresponds to a year. Mean temperature was calculated using the time series for each province (extracted from the gridded GHCN CAMS dataset), and taking the mean of the mean temperatures across provinces and months. The colours of the points indicate the years, going from the earlier part of the time series (dark colours) to closer to present (lighter colours). The Pearson correlation between ln dengue cases and temperature is 0.53. The underlying data are in S13 Data at `https://github.com/UF-IDD/synchrony_dengue_figures`.

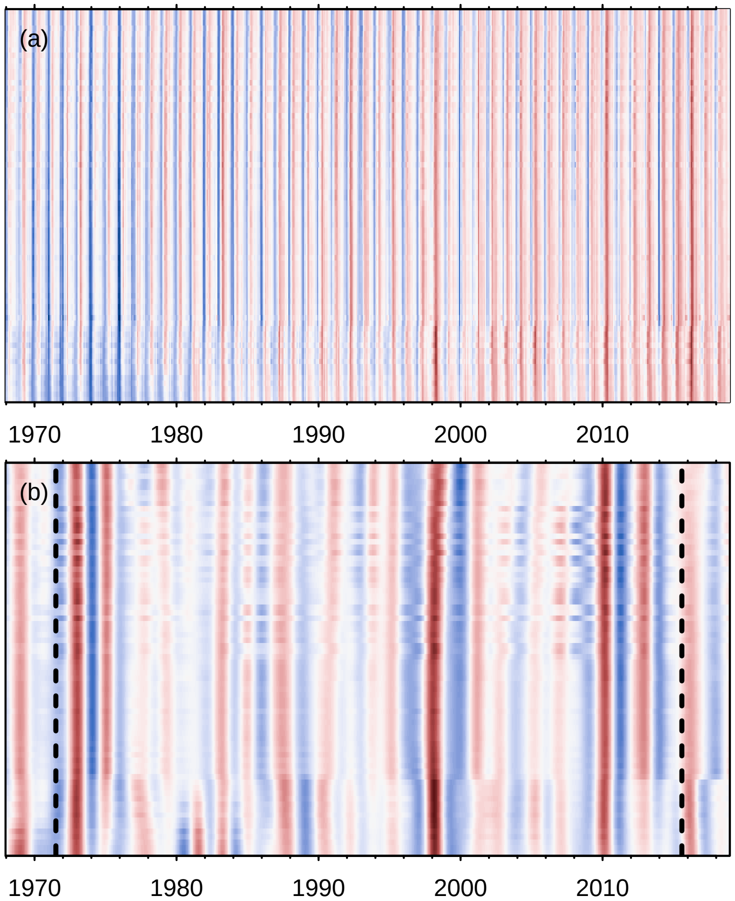

Fig S37. Heatmaps of mean temperatures. Heatmaps of (a) mean temperatures and (b) reconstructions of temperature time series using multiannual components only, per province arranged from north (top) to south bottom. To improve clarity, values for each province were normalised to a mean of zero and standard deviation of one ($\mu = 0, \sigma = 1$) in all panels. Blues (respectively reds) are lower (respectively higher) temperatures, and whites correspond to zero. Edge effects in the wavelet transforms may influence results before and after the vertical dashed lines in (b). The underlying data are in S13 Data at `https://github.com/UF-IDD/synchrony_dengue_figures`.

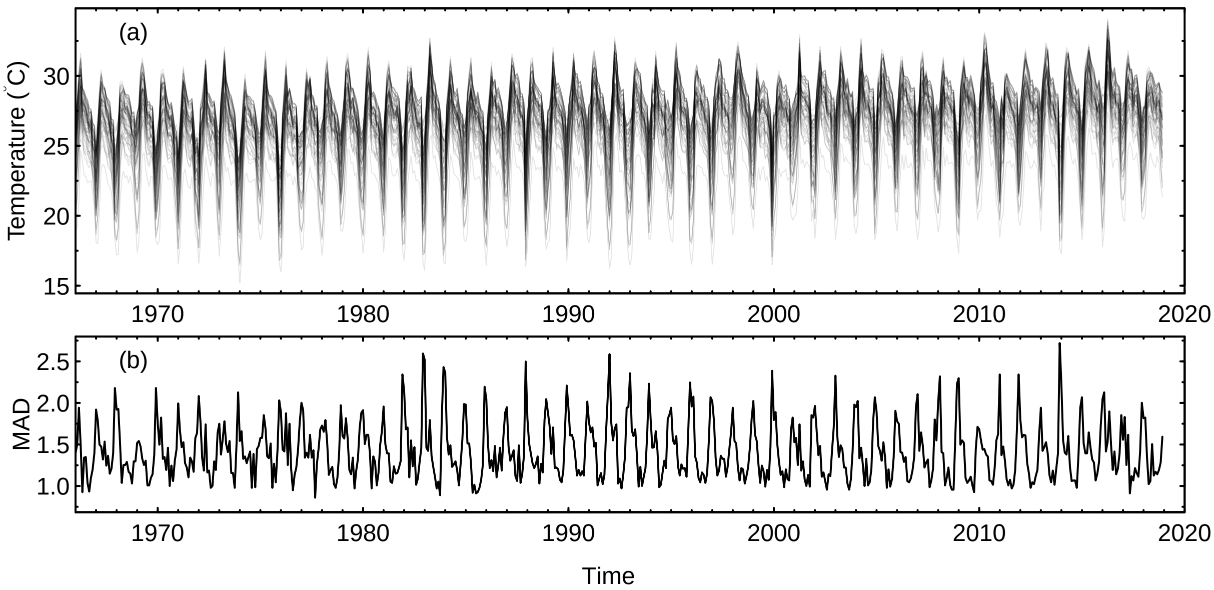

Fig S38. Mean temperature time series for all Thai provinces. (a) Mean temperature time series for all provinces, and (b) the median absolute deviation (MAD) across all provinces, for each point in time. The pattern in the MAD shows that winter temperatures are distinctly more variable across the country than the summer temperatures, but temperatures are not more similar across the country during synchronous events (e.g., between 1998 and 2002). The underlying data are in S13 Data at `https://github.com/UF-IDD/synchrony_dengue_figures`.

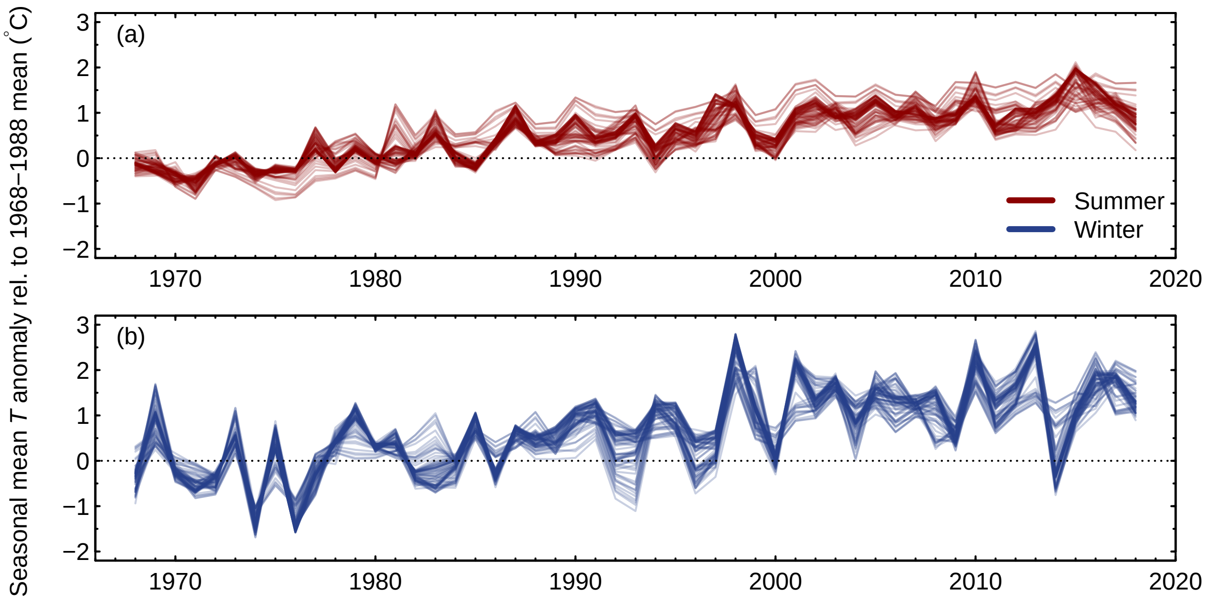

Fig S39. Seasonal mean temperature anomalies relative to the seasonal means during 1968–1988, for (a) summer mean temperatures, and (b) winter mean temperatures, for each province. I.e., a value of zero in panel (a) means that the mean summer temperature for that year was approximately equal to the mean temperatures during summer months between 1968 and 1988. The underlying data are in S13 Data at `https://github.com/UF-IDD/synchrony_dengue_figures`.

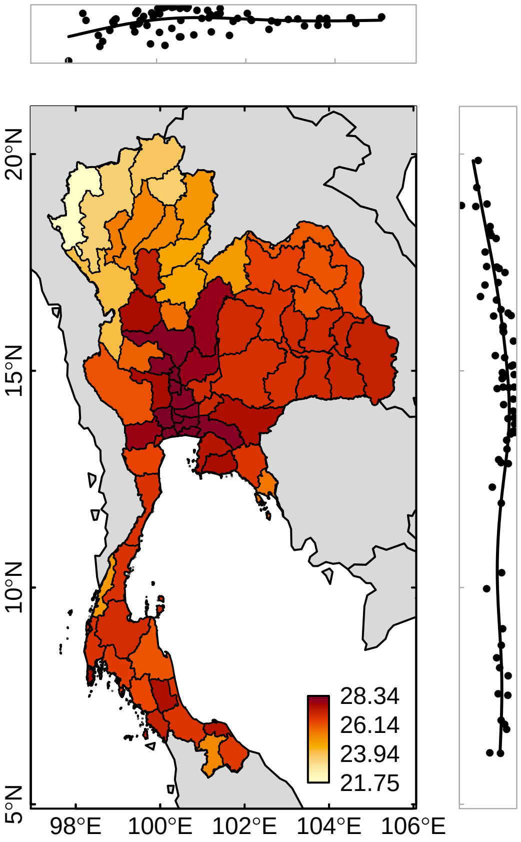

Fig S40. Mean temperature map for each province across the entire time series (1948–2018). Side panels show the mean temperatures as functions of latitude and longitude, and the line is a generalised additive model (GAM) fit to the points. Thai administrative boundaries were downloaded from `https://data.humdata.org/dataset/thailand-administrative-boundaries`, and those for the neighbouring countries were downloaded from `http://thematicmapping.org/downloads/world_borders.php`. The underlying data are in S13 Data at `https://github.com/UF-IDD/synchrony_dengue_figures`.

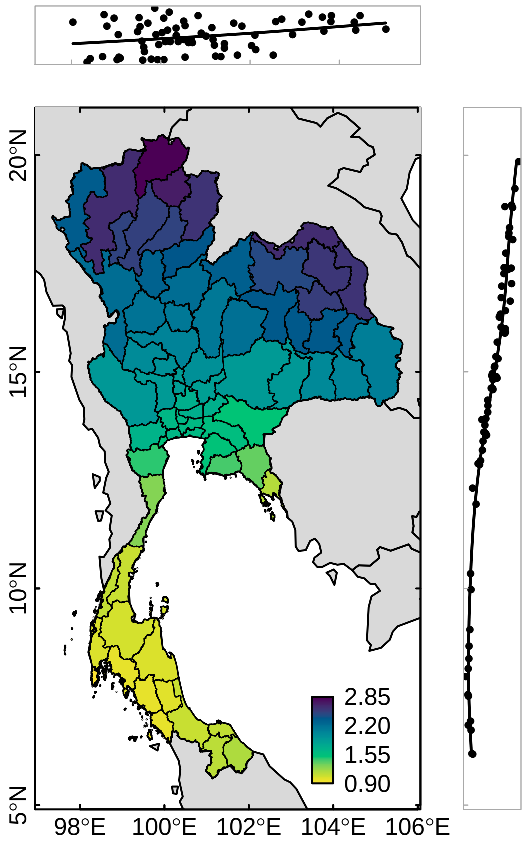

Fig S41. Standard deviation of temperature for each province across the entire time series (1948–2018). Side panels show the standard deviation of temperatures as functions of latitude and longitude, and the line is a GAM fit to the points. Thai administrative boundaries were downloaded from `https://data.humdata.org/dataset/thailand-administrative-boundaries`, and those for the neighbouring countries were downloaded from `http://thematicmapping.org/downloads/world_borders.php`. The underlying data are in S13 Data at `https://github.com/UF-IDD/synchrony_dengue_figures`.

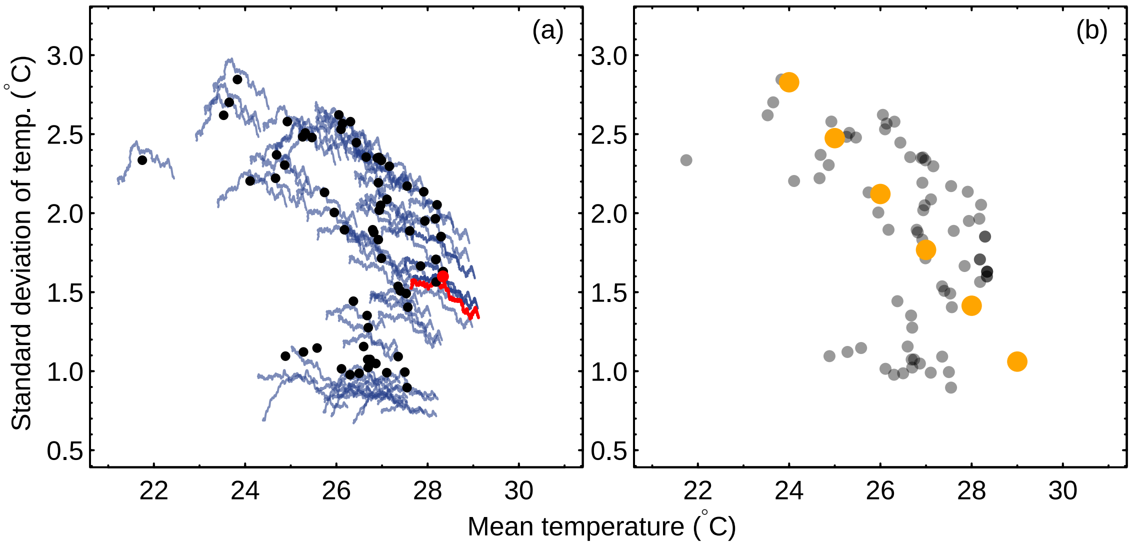

Fig S42. Patterns in temperature across provinces. (a) Mean temperature vs. standard deviation of temperature estimated across the entire time series (1948 – 2018) for each province (black points), and for a rolling 25-year window (blue lines). The red point and line highlight Bangkok. The directions of the lines are all from left to right (i.e., mean temperatures increase over time). (b) In black, the same points as in (a), and overlaid in orange are the parameters used for the transect of six hypothetical locations used in simulations. The underlying data are in S13 Data at `https://github.com/UF-IDD/synchrony_dengue_figures`.

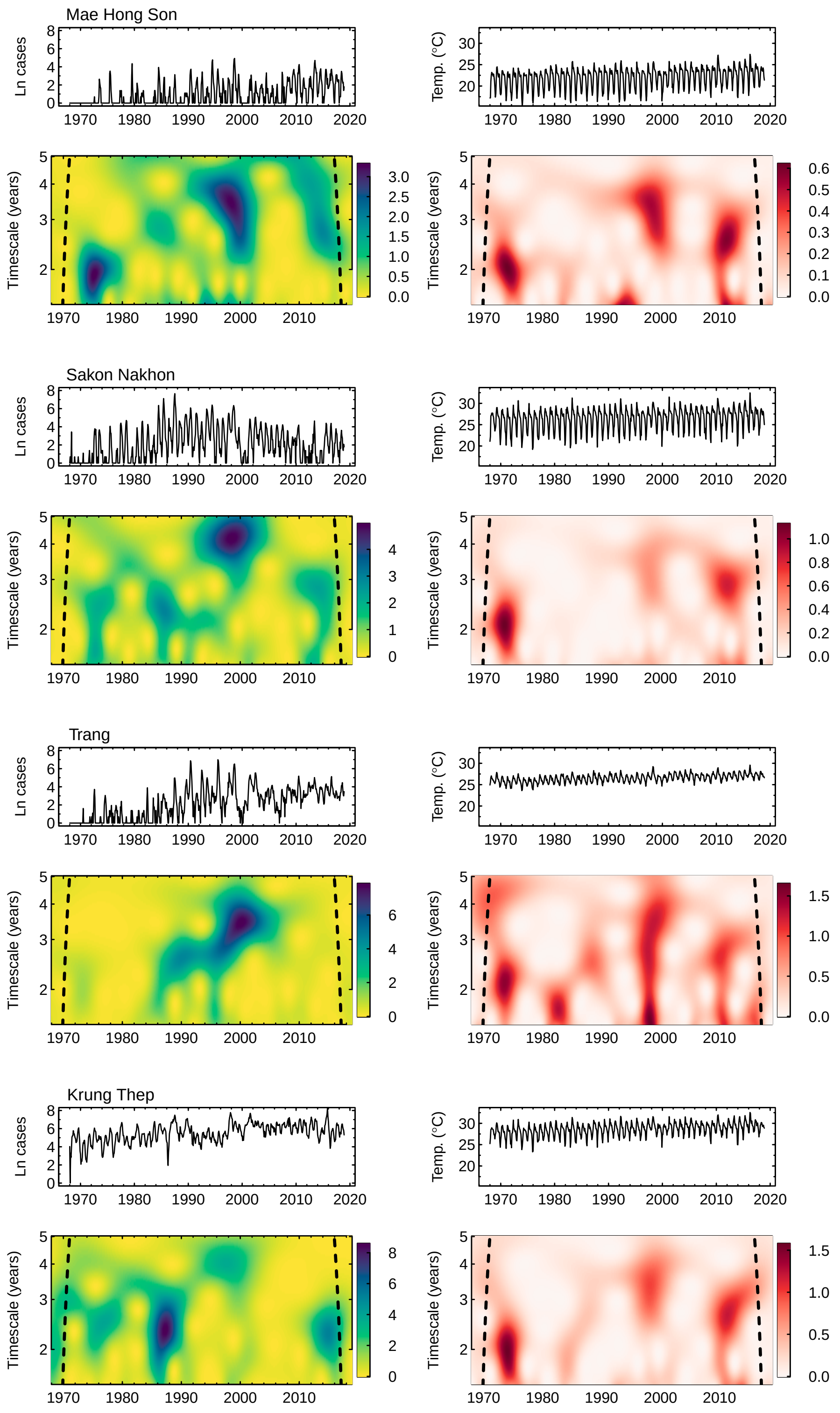

Fig S43. Temperature, dengue cases, and respective wavelet spectra for four example Thai provinces. For four example provinces in Thailand with different thermal regimes, the top left panels show ln number of dengue cases (and the name of the province), the top right panels show the mean monthly temperature, and the panels below show their respective wavelet spectra (for multiannual timescales only, for clarity). The wavelet spectra for temperature are for the nonlinearly detrended temperature time series. The underlying data are in S13 Data at `https://github.com/UF-IDD/synchrony_dengue_figures`.

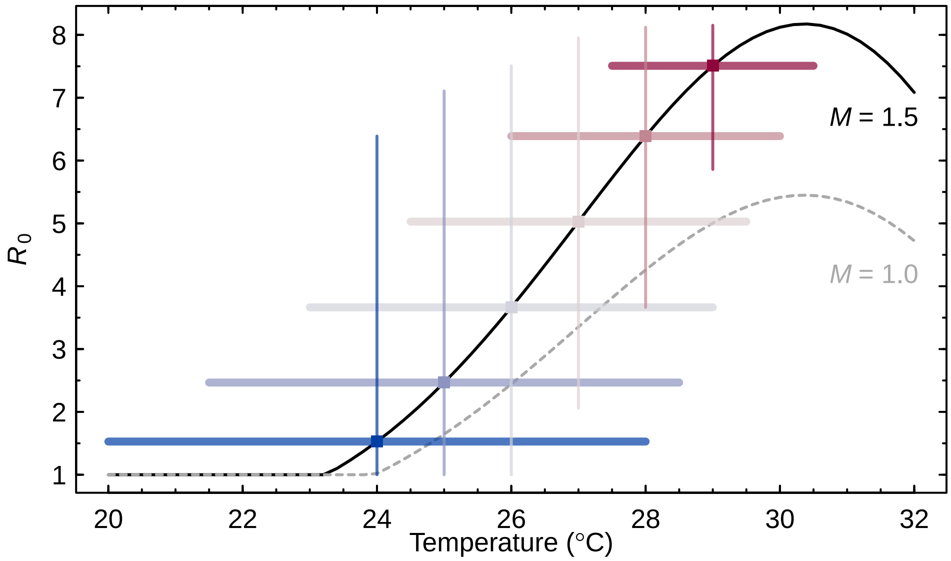

Fig S44. Temperature dependence of R_0, for two different ratios of mosquitoes to hosts (M), where the coloured points and horizontal lines indicate the mean and amplitude of the synthetic temperature time series for the six hypothetical locations used in simulations (corresponding to the orange points in Fig S42b). The vertical lines show the corresponding range of transmission experienced in each location. Note how as the mean temperature increases, the amplitude decreases, which combined with the fact that the slope of the response function decreases at higher temperatures, results in a lower variation in transmission. R_0 values were estimated by running a single-serotype version of the model across a range of mean temperatures until equilibrium, and calculating the inverse of the susceptible proportion of the host population. The underlying data are in S13 Data at `https://github.com/UF-IDD/synchrony_dengue_figures`.

References

1. Mordecai EA, Cohen JM, Evans MV, Gudapati P, Johnson LR, Lippi CA, et al. Detecting the impact of temperature on transmission of Zika, dengue, and chikungunya using mechanistic models. PLOS Neglected Tropical Diseases. 2017;11(4):e0005568. doi:10.1371/journal.pntd.0005568.

2. Cator LJ, Johnson LR, Mordecai EA, El Moustaid F, Smallwood TRC, LaDeau SL, et al. The Role of Vector Trait Variation in Vector-Borne Disease Dynamics. Frontiers in Ecology and Evolution. 2020;8. doi:10.3389/fevo.2020.00189.

3. Barbazan P, Guiserix M, Boonyuan W, Tuntaprasart W, Pontier D, Gonzalez JP. Modelling the effect of temperature on transmission of dengue. Medical and Veterinary Entomology. 2010;24(1):66–73. doi:10.1111/j.1365-2915.2009.00848.x.

4. Huber JH, Childs ML, Caldwell JM, Mordecai EA. Seasonal temperature variation influences climate suitability for dengue, chikungunya, and Zika transmission. PLOS Neglected Tropical Diseases. 2018;12(5):e0006451. doi:10.1371/journal.pntd.0006451.

5. Palamara GM, Childs DZ, Clements CF, Petchey OL, Plebani M, Smith MJ. Inferring the temperature dependence of population parameters: the effects of experimental design and inference algorithm. Ecology and Evolution. 2014;4(24):4736–4750. doi:10.1002/ece3.1309.

6. Lambrechts L, Paaijmans KP, Fansiri T, Carrington LB, Kramer LD, Thomas MB, et al. Impact of daily temperature fluctuations on dengue virus transmission by *Aedes aegypti*. Proceedings of the National Academy of Sciences. 2011;108(18):7460–7465. doi:10.1073/pnas.1101377108.

7. Carrington LB, Armijos MV, Lambrechts L, Scott TW. Fluctuations at a low mean temperature accelerate dengue virus transmission by *Aedes aegypti*. PLoS Neglected Tropical Diseases. 2013;7(4). doi:10.1371/journal.pntd.0002190.

8. Brady OJ, Johansson MA, Guerra CA, Bhatt S, Golding N, Pigott DM, et al. Modelling adult Aedes aegypti and Aedes albopictus survival at different temperatures in laboratory and field settings. Parasites & Vectors. 2013;6(1):351. doi:10.1186/1756-3305-6-351.

9. Bütikofer L, Anderson K, Bebber DP, Bennie JJ, Early RI, Maclean IMD. The problem of scale in predicting biological responses to climate. Global Change Biology. 2020;26(12):6657–6666. doi:10.1111/gcb.15358.

10. Johansson MA, Powers AM, Pesik N, Cohen NJ, Staples JE. Nowcasting the Spread of Chikungunya Virus in the Americas. PLOS ONE. 2014;9(8):e104915. doi:10.1371/journal.pone.0104915.

11. Bjørnstad ON, Ims RA, Lambin X. Spatial population dynamics: analyzing patterns and processes of population synchrony. Trends in Ecology & Evolution. 1999;14(11):427–432.

12. Bjørnstad ON. ncf: Spatial Nonparametric Covariance Functions; 2009.
