# Supplementary material for: Periodic synchronisation of dengue epidemics in Thailand over the last 5 decades driven by temperature and immunity
Source: PLoS Biol. 2022 Mar 18;20(3):e3001160. doi: 10.1371/journal.pbio.3001160 (PMC8967062; doi:10.1371/journal.pbio.3001160)
Supplement: Attachment — Submitted filename: Garcia-Carreras_et_al_response_to_reviewers.pdf [file pbio.3001160.s002.pdf]

Response to reviewers

We would like to thank both reviewers for their thoughtful and insightful reviews. They raised very good points, and in addressing them, we think our manuscript is substantially improved. Below, we address each of the points raised by the reviewers and address them one by one in blue text.

Reviewer 1

In this work the authors examine the synchrony of reported cases of dengue (for Dengue Hemorrhagic Fever, or DHF) across Thailand with and unprecedented data set spanning 51 years across 72 provinces. The synchrony of temperature and that of temperature with dengue cases are also addressed. A process-based model for the population dynamics of dengue driven by temperature is used to examine the role in the generation of multiannual cycles of extrinsic forcing (by temperature) vs. that of intrinsic dynamics (through cross-protection between different serotypes).

The appeal of this study is the spatio-temporal data set, which provides a longer window of time to address the role of climate vs. immunity in the population dynamics of endemic dengue across this large region. Previous studies had already examined a role of climate drivers and synchrony, in particular with reference to the El Niño Southern Oscillation (ENSO). Here, a longer perspective becomes possible. Indeed, the patterns of Figure 1 are interesting.

Unfortunately, there are substantial issues with the logic and results of the analyses. Results do show that dengue in Thailand moves in an out of synchrony, with periods of widespread and significant synchrony. The evidence for a role of temperature on these patterns is fairly convincing, except for some inconsistencies raised below. The evidence for a role of immunity, through cross-protection, is not convincing and remains confusing in its argument.

My major comments below explain why. They also indicate my concerns with the evidence developed to explain the patterns of synchrony. The study in its current form is incomplete. The statistical and modeling machinery exists today to address different hypotheses on the synchrony, by fitting to these time series process-based models and applying model selection. I am not asking the authors to go that way, but a better comparison of the modeling exercise to the

data, and a clearer reasoning on the role of cross-protection is needed.

We agree that our manuscript had some gaps in logic and thank the reviewer for pointing these out. Below in responses to specific critiques we point to changes in the manuscript that we have made to address these gaps. We have also explained our rationale for the modeling work and included more direct evaluation of modeling results with data. Specific changes are described below.

Major comments:

1. Role of intrinsic dynamics vs. extrinsic factors: The argument is particularly confusing. The simulations (number 3) include multiannual variation in the synthetic "temperature" forcing (basically a sinusoidal modulated in its amplitude), and then compare the mean wavelet power of the response at the temporal scale of the forcing to the mean wavelet power at the dominant temporal scale of what is considered the "intrinsic" dynamics. The latter is obtained from the same model forced by the seasonal synthetic temperature with no multiannual variation. In other words, the comparison is for the intensity of the multiannual response with and without the multiannual variation in the synthetic temperature (and at different time scales since the dominant multiannual periods differ for those two runs). We are shown that the power of the multiannual variation caused by the "intrinsic" cycles increases for longer duration of cross-protection and higher mean temperature (with lower variance). Why does this imply that immunity is needed to explain the observed patterns of synchrony? The logic is unclear and possibly flawed. We expect higher mean temperatures (if they cause higher transmission intensity and are closer to the optimum for transmission) to make the role of immunity more prominent as the nonlinear dynamics of the disease becomes more important. This does not tell us that the multiannual variation and its patterns of synchrony in the observed cases necessitate a role of cross-protection. The characteristic multiannual time scale generated in this way differs from that in the temperature forcing. Is this the case in the data? Moreover, since the variability of temperature involves multiple time scales (in particular, seasonal and multiannual), it would be important to provide a convincing argument for why it is not a sufficient explanation for the patterns observed in the data. That intrinsic dynamics (just with seasonal forcing) produces under the longest cross-protection considerable interannual variability with a characteristic period shorter than 4 years is not per se evidence that such cross-protection is important in the real system.

Our argument with regards to immunity and cross-protection was not originally intended to hinge on proving that it is essential to explain the observed patterns in synchrony. Having established that temperature plays an important role in shaping the observed patterns in dengue synchrony (at least during synchronous events; see below), we were mostly interested in understanding how these patterns might depend on and vary with the mean duration of cross-protection, because other work has found immunity to be an important element of the dengue

system [1, 2, 3]. What we found, with simulation 3, was that a single common multiannual cycle was sufficient to synchronise an otherwise asynchronous system (as established in simulation 2), but the extent to which it could synchronise dengue dynamics did depend on the duration of cross-protection, with longer cross-protections leading to weaker synchronisation. Thus, rather than comparing two hypotheses: extrinsic vs. intrinsic or intrinsic with and without cross-protection, we meant our simulations to explore the filter that possible intrinsic mechanisms could provide in understanding impacts of temperature on dengue and to more generally explore the mechanism by which temperature increases could drive synchrony given our knowledge of other aspects of the dengue system. Our empirical evidence provides support for the importance of temperature and previous work (cited above) provides evidence that immunity is important in this system. Here in our simulations, we intended to explore their interplay through multiple model formulations.

However, we do acknowledge that our treatment of immunity may have been confusing. We did also present results, those from simulation 1, as providing some support for the importance of cross-protection in shaping the observed patterns. In our results, the correlation between the modelled dengue synchrony and observed dengue synchrony was statistically significant across a range of cross-protections. On the other hand, the correlation between observed dengue synchrony and the synchrony of the temperature variable itself was not statistically significant. We also implied that immunity is the main mechanism at play when explaining periods of asynchrony (simulation 2), but, as you point out, asynchrony in dengue may have come about through asynchrony in temperature multiannual cycles and not intrinsic dynamics. The cross-wavelet mean fields (CWMFs) we present cannot really distinguish between these alternative explanations.

To address this, we have changed the heading of this section from "Simulations to disentangle the roles played by temperature and immunity" to "Simulations to explore mechanism: the interplay of temperature and immunity". We also provide a better explanation of the patterns in synchrony we observe through additional analyses. Using the dengue data wavelet mean fields (WMF), we defined points in time and timescales (henceforth, "conditions") above the 75th percentile of WMF values as "high synchrony" and those below that value as "low synchrony" (see the new Fig S15 in SI, in which we also show alternative choices of percentiles). Then we examined the local variability (including unsynchronised wavelet content) in each of the provinces under these two conditions. For each province, we calculated the Spearman correlation between the square-amplitudes of the wavelet spectra of dengue (data) and temperature (data), separately for high and low-synchrony conditions, yielding two correlation coefficients (r values) per province. We established that temperature was the main driver during synchronous events (with our analysis using the CWMF), suggesting we should expect statistically significant positive r values between temperature and dengue square-amplitudes in high-synchrony conditions. Were temperature to also have

been the main driver in the asynchronous conditions, we might expect the r values in low-synchrony conditions to be similar to the r values in high-synchrony conditions. Low or no correlations would indicate either that intrinsic dynamics play a greater role during asynchrony, or that temperature and dengue have little multiannual dynamics at those times and timescales. Our results show that r values are statistically significantly higher in high-synchrony conditions than in low-synchrony conditions (see Table S2). Correlations for low-synchrony conditions were significantly greater than zero, but comparatively low. However, this result alone is not sufficient to establish that intrinsic factors play a greater role during asynchrony. Next, we performed the same analysis, but comparing the wavelet spectra of modelled dengue output and dengue data. Here, the correlations in high-synchrony conditions were also significantly greater than during low synchrony, but the differences in the correlations between the two conditions were on average smaller (see Table S2). These results show that the model captures some of the mechanisms at play in each province during low synchrony, and explains some of the variation that temperature alone cannot. Both these results therefore at least support the hypothesis that intrinsic dynamics play a more important role during these asynchronous periods. We have added Fig S16 in SI summarising some of these results. Results were found to be robust to the choice of percentile defining high and low synchrony conditions (Figs S17 and S18).

We have incorporated these analyses and their corresponding methods into the main text.

Comparisons of these simulations to the empirical patterns are needed, rather than between simulations themselves. I would have liked to see the actual patterns of variation in dengue cases as a function of latitude, for representative provinces of the temperature "regime". Do the predictions of Figure 4 c better explain the data than those of Figure 4b? Do we see periods in the dengue dynamics that are not present in the temperature?

Fig 1 shows multiannual patterns ordered by latitude, and in response to a point made below, we have also added Fig S43 in SI showing time series and wavelet spectra for example provinces in Thailand.

The purpose of simulations 2 and 3 was not to directly compare results with empirical patterns; that was the intended purpose of simulation 1, where we used the actual temperature time series to drive the model. There, we make explicit comparisons between the patterns in synchrony produced by the model with those observed empirically using correlation coefficients. These statistics transparently test the extent to which global patterns in synchrony produced by the model resemble the observations. We also now look at correlations between dengue model output and dengue data for high and low-synchrony conditions (see our response to the point above). With simulations 2 and 3 we aimed to under-

stand the mechanisms that could produce the observed periods of asynchrony and synchrony, respectively. As we explain in response to a specific question on this below, we used simplified synthetic time series of temperature so that we could be sure that, in absence of multiannual frequencies in temperature, multiannual frequencies in dengue could be ascribed to intrinsic dynamics. The patterns seen in Figs 4(a–c) are therefore idealisations. The dengue data will predictably have a mix of different processes, including times when multiannual cycles are driven by temperature (particularly during periods of synchrony), and times when they perhaps are determined, to a greater extent, by intrinsic dynamics. By looking at time-averaged wavelet power, all these processes would be mixed, and the expectation that we might observe systematic patterns such as those in Fig 4(a–c) would be overly optimistic.

The power at a given multiannual frequency is not the only aspect of the synchrony that matters; when in time those cycles occur is also important and highly non-random here (see 2 below). One may get multiannual cycles out of "intrinsic" dynamics , even cycles with periods observed in the data, but their timing will not be synchronous across the region without multiannual variation that is extrinsic setting the timing.

We agree. In fact, the CWMF also accounts for the consistency of phases between dengue and temperature across locations (see section "Materials and methods", subsection "Drivers of synchrony"). If different locations were to have significant wavelet power at some multiannual timescale at a point in time, but this power were to be mainly ascribed to intrinsic dynamics, it is unlikely that the phases with temperature would be consistent across locations, and so the CWMF would be low(er). Indeed, there are many points in time when the CWMF is relatively low (Fig 2a-c). High CWMF values shown in Fig 2 imply similar phases across locations, particularly during synchronous events, and thus point to temperature as the main driver of dynamics at those points in time.

(On a more technical note, I could not tell whether in the comparisons of Figure 5 the multiannual variation in the synthetic temperature was introduced for a single such cycle like in Figure 4e).

As mentioned in Table 1 in the main text, the seasonal sinusoids have "a superimposed single multiannual fluctuation", as in Fig 4e. We now also specify a "single environmental multiannual fluctuation" in the caption to Fig 5.

2. Climate variability as an explanation for the patterns: By eye, the patterns of Figure 1 are very suggestive of a role of El Niño events in the synchrony patterns. The years when cases are high across Thailand have a high correspondence to some major EN events, namely 1986-87 and 1997-1998. This is consistent with

previous studies of dengue dynamics in the region although those studies were more limited in the data. I appreciate the consideration of regional climate (here, temperature) but it seems surprising and incomplete to leave out of an explanation for the synchrony how ENSO is manifested in the regional climate and how this manifestation has changed across events. ENSO is a major driver of the regional climate in Thailand and the way it affects temperature (and also rainfall) should be relevant to the observed patterns, especially when acting on top of the long-term warming trend.

We did not focus on ENSO because our aim was to explore whether the patterns in synchrony of dengue we observed could be ascribed to an environmental variable we know directly affects the vector of dengue, thus allowing us to draw a mechanistic link. ENSO would only be invoked to explain the patterns in synchrony observed in temperature, which was beyond the scope of our manuscript. Nevertheless, we agree that ENSO needs to be discussed as the driver of those patterns in temperature. We have added a paragraph centred around ENSO in the Discussion.

3. Inconsistencies between patterns of synchrony in cases and temperature, and between those in observations and simulations: There are some convincing similarities between patterns of synchrony in temperature and cases. There are some exceptions to this correspondence that should be explained in some way. Namely, the synchronous event in early 1970s which is present in the temperature but not in the cases. Because it is present in the temperature, it also shows up in the simulated cases (Figure 2 c). Again, this differs from what is observed in the data. Similarly, the cross-wavelet mean fields in panels (d) and (e) differ for the simulations and the data. Could there be an effect of initial conditions? Of reporting rates in that first part of the data? Of the long-term trend in temperature? Another inconsistency is the non-significant correlation between the wavelet synchrony of dengue and that of temperature, compared to the significant correlation of the same quantities for the simulations. Lines 303-306 argue that this discrepancy highlights the importance of nonlinearities. I do not follow the argument: the nonlinearities should affect both model and observations.

As we mention in the Discussion, we hypothesise that the inconsistency in the early 1970s is likely due to reporting rates during that period (but see our response to a similar comment by Reviewer 2 below).

With regards to the correlations, we think the confusion is due to a misunderstanding. Our argument was that the correlation between the temperature (data) and dengue (data) WMFs was not significant, but that between the dengue (data) and dengue (model output) WMFs was (so not comparing the same quantities as such), indicating that the various nonlinearities encoded in the model, together with cross-protection, translated the temperature WMF into something that statistically looked more similar to observed patterns in dengue.

4. Better understanding of intrinsic dynamics (with just seasonal forcing); In the population dynamics of infectious diseases, the multiannual variation that is called intrinsic arises from seasonality interacting with the natural (damped or persistent) oscillations caused by the nonlinear dynamics of the disease itself. Here, there are some confusing statements about multiannual cycles arising in part because seasonality itself becomes less important. This is not a given. Seasonality is an integral component of generating the interannual variability. Whether or not weaker seasonality is required we cannot tell, as the mean of temperature grows at the same time that seasonality weakens. Generalizing from such limited simulations seems incomplete. For warmer temperatures and higher transmission, the depletion of susceptibles and effects of cross-protection should also grow.

We initially were trying to simplify the problem and focus on the five (now six; see our response to the comment on R_0 values below) hypothetical locations that were roughly representative of climatological patterns present in Thailand. However, we (think we) understand your point here, and broadly agree. We have extended the analyses for Fig 5 in the main text to include the other combinations of mean temperatures and seasonal amplitudes, to provide a more complete picture. The results show that, indeed, if seasonal variation is large enough, even at high mean temperatures and with long cross-protections, temperature may still drive multiannual dynamics in dengue. This temperature regime, however, is not typically observed in Thailand.

5. The parameterization of the carrying capacity was difficult to follow, in terms of the value reported in Huber et al. and the one used here. It should be possible to clarify with the authors whether there was a reporting mistake in that paper and why such a different value is required here. The carrying capacity is a very challenging aspect of dengue models where the dependency on rainfall should show up. It is difficult to follow that part of the model and even harder to understand the need for such a different parameter. It would be important to clarify this so that the confusion on this matter does not propagate in the literature.

We agree that the carrying capacity formulation is tricky and somewhat confusing. We could have clarified with Huber et al. on what value they actually used, but we did not because we found the evidence and justification for using any one particular value to be very weak, so we would in any event have had to perform robustness tests on that parameter (Huber et al. textually state that the activation energy was set to 0.5, "a conservative estimate as we lacked sufficient data on estimates of the carrying capacity of Aedes aegypti and its underlying temperature dependence."). Clarifying what value was used by Huber et al. could be counterproductive and in fact have the risk of misrepresenting the

state of knowledge of what this value should be. Instead, we think this question of density dependence and carrying capacity in mosquito populations and how they vary with temperature really merits further research. We make this point in section "Caveats on the dengue model" in SI, which we now refer to from the main text.

The following comments are not major ones; they are needed to clarify the work.

6. Why was the trend removed for the cases but not temperature?

We removed the trend from temperature, not cases. The reason for removing the trend is to focus on the oscillatory behaviour, and not long-term trends that will only inflate low-frequency signals. However, we did not remove the trend from cases because time series contain zeroes, and, as we explain in section "Materials and methods", subsection "Wavelet transforms", ln-transforming these and removing a trend produces artificially odd time series (see example in Fig 1 below).

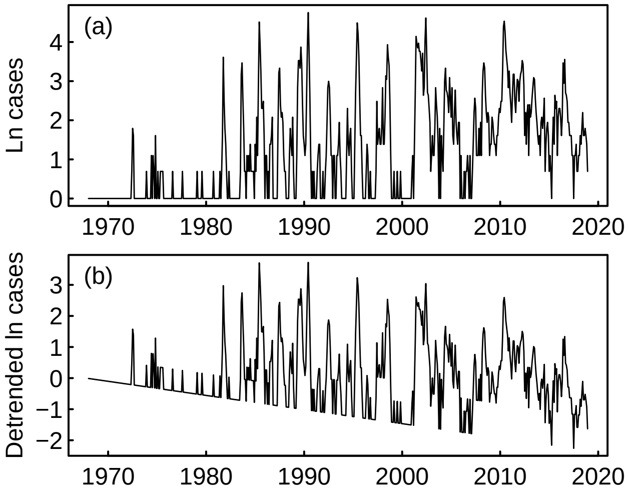

Figure 1: Example detrending of a time series. (a) Time series of ln-transformed number of cases for an arbitrary Thai province. (b) The same time series as in (a) having removed a linear trend. Note how particularly at the beginning of the time series, due to the presence of zeroes, an artificial sloping line (the zero values in (a)) is produced as a result of detrending.

7. The statement that endemic regions may be those most strongly affected by climate change is not that meaningful without the context of the range of the reproductive number of the disease and its dependence on temperature. The

effect of climate will depend on where a region lies in this dependence. This is so for the same reason that the model used here shows a weaker sensitivity to temperature forcing where the function of the transmission rate with temperature is closer to the optimum.

This sentence was not worded carefully. We did not intend to say that endemic regions will be most affected by climate change, because the sensitivity of dengue to climate there is greatest. In fact, we mean that the burden of dengue is largest in areas where dengue is already endemic, and thus impacts of climate change may have large effects on dengue burden given that any variation in transmission due to climate change might be expected to affect an already very substantial incidence. However, we agree that it is not clear and even our intended meaning is speculative. We have edited the sentence to just focus on the lack of work to estimate the effects of climate change in endemic areas. We have changed this text to read: "However, less work has been done to estimate the effect of climate change on dengue in regions where the pathogen is already endemic."

8. It would be useful to provide the R0 (or vectorial capacity of the model) as a function of the relevant range of temperatures. The figure of the biting rate and infectiousness with temperature does not consider all the parameters that depend on temperature and affect R0 in the model.

Thank you for the suggestion. We have now replaced the Figure with one showing R_0 as a function of temperature (Fig S44 in SI). Indeed, in addressing this question, we realised we were running the model with R_0 values that were higher than might have been expected for dengue in Thailand, so we have now reproduced all analyses with R_0 values closer to our expectation, and added text in section "Materials and methods", subsection "Temperature-dependent dengue model" in the main text explaining our choice. We also redefined the hypothetical locations we use for simulations 2 and 3 so they are now six (instead of five), and these now more closely reflect observed thermal regimes in Thai provinces (Fig S42 in SI). Our results and conclusions have not qualitatively changed as a result of these changes. We have also added results in section "Robustness tests" (Figs S5 and S6 in SI) for alternative (lower still) values of R_0 (the curve corresponding to a ratio between mosquitoes and human hosts $M = 1$ in Fig S44 in SI). Results proved to be robust to the lower R_0 values.

9. Why use a sinusoidal for the synthetic temperature and not just the seasonality of empirical temperature in a given location? (By seasonality, I mean here the mean temperature pattern as a function of time of the year). It is possible that a sinusoidal fits this pattern well, in which case such a fit could be used.

The reason was to simplify the problem to the extent possible. Using a sinusoidal function means that the temperature input contains one single frequency, so we

can then be sure that any other frequencies detected in the output are for reasons other than multiannual frequency content of temperature. Empirical seasonality invariably would have incorporated further frequencies. We have added text to section "Mechanistic dengue model and simulations" to clarify this.

10. I would have liked to see representative plots of the time series of cases across the region, especially for those locations that are chosen for the different temperature regimes. Similarly, it would be informative to see the wavelet spectra of these time series.

Thank you for the suggestion. We have added Fig S43 in SI showing time series and wavelet spectra for both dengue and temperature, for four provinces across Thailand. These are representative of different thermal regimes in Thailand. The spectra focus on the multiannual timescales only for clarity (the signals, for both temperature and dengue, are dominated by the seasonal signal, sometimes making it difficult to see the multiannual patterns).

11. Please provide some information on the spatial resolution of the temperature data compared to the resolution of the dengue data set.

As mentioned in section "Materials and methods", subsection "Data", the spatial resolution of the temperature dataset is $0.5° \times 0.5°$*, where we use the grid point closest to the centroid of each province as the temperature time series for that province. Dengue time series are given per province (where the size of provinces can differ substantially).*

12. Figure 2: I would have liked to see significance on the wavelet mean-field plots, although I was unclear on whether this can be done, as the significance appears to be evaluated with a different quantity that concerns the phases.

We went to great lengths to ensure that our measure of significance was not biased in any way. We understand that what you mean here is that you would have liked to see a contour overlay of the significance on the WMFs. This would require a point-by-point significance test, which would lead to substantial non-independent multiple testing of all the points in the matrix and some features would inevitably appear significant for both real and spurious data. We were specifically interested in knowing at which points in time, rather than at what multiannual timescales, synchrony was significant, which led us to the multi-step approach outlined in section "Materials and methods", subsection "Wavelet mean fields as a measure of synchrony", where we make comparisons based on slices in time averaged across timescales.

As to why we use the phase component only to estimate significance, the rea-

son derives from the choice of working with slices in time. The WMFs are normalised so that the time average (RMS) at any timescale has a maximum value of 1, if the wavelet components drawn from each location have matching phase and amplitude dynamics. This normalisation allows comparison of timescale-dependent time average synchrony values, and straightforward significance testing of the time averages. However, when averaging across timescales the WMF values at a given time are not bounded in this way, as all the wavelet amplitudes at a particular time may be much higher than their time average. Times when wavelet amplitudes are above average are likely to have high WMF values whether or not the transforms from different locations are in phase, potentially implying a different null hypothesis at each time and/or introducing ambiguity about the meaning of surrogate testing results. Instead we elected to use significance testing of the phase information only, to pick out times when the transforms were in phase, associated with features appearing in both the WPMF and WMF. The WPMFs only retain information on the phases, and are thus not affected by the issues around amplitude normalisation. The same statistical test can be applied across times and timescales to identify times of high synchrony. In general, these times are also associated with high wavelet amplitudes, enhancing the prominence of the synchronised features as they appear in the WMF.

We have now incorporated this explanation into section "A note on estimating significance of (cross-)wavelet mean fields" in SI, and refer to this section in the main text.

13. What is shown in Figure 3 (a)? The caption was unclear about this.

Fig 3a shows exactly the same curves as in Fig 3b, but overlayed. As the caption states: "The two panels show the same time series, but (b) separates them for clarity".

14. I was not convinced by the value of examining the periodicity of the synchrony. What does this tell us given the low number of synchrony cycles, and given patterns dominated by two main events of large synchrony (corresponding to particular El Niño events about 12 years apart)?

We provided this analysis mainly because the degree of synchrony did appear to be periodic, even despite having few cycles. We also did not intend to place too much emphasis on this result for the reason you mention, which is why we kept this section short.

15. Line 140: clarify how the normalization is performed.

We normalised the time series such that the resulting time series had a mean

of zero and a standard deviation of one. We have now clarified this in the text too.

16. The abstract would benefit from a complete rewrite of the part that states the findings.

In light of the points raised above, we have reorganised and partly rewritten the abstract, as suggested.

17. In general, I found the definitions of the different quantities related to wavelets somewhat difficult to follow, especially in places where significance is tested on one quantity but synchrony is evaluated with another. I know the references are provided for the methods. It would be useful anyway to have a more self-contained set of definitions that a general reader can follow here.

The references provide greater details for some of the methods we use (particularly wavelets and WMFs), but section "Materials and methods" contains all the definitions and information used in the manuscript. We developed the approach to estimate significance ourselves, and that differs from approaches used in the references we cite. We understand that, in particular, the fact that synchrony is presented using one quantity (sensitive to amplitude variation and phases) but significance on another (sensitive to phase synchrony only) may be confusing. We outlined the reasons in a response above. We have now added text explaining our rationale in the SI section "A note on estimating significance of (cross-)wavelet mean fields".

Reviewer 2

The authors use a long-term spatiotemporal data set of dengue incidence in Thailand to investigate synchrony in outbreaks across provinces through time. They find that there have been multiple synchronization events, and then use a mechanistic model to illustrate how this pattern can be driven by both temperature and immune dynamics.

I think this is an exciting and thorough study on a topic that should be of wide interest. I also agree with the authors that studying how climate affects infectious disease in endemic locations is relatively understudied compared to the research focus on disease range expansions and shifts. I have three "major" comments on potential ways to improve the manuscript for a wider audience such as PLOS Biology's.

Major Comments:

1. After the relatively long Results section, I found the Discussion to be short and a bit underwhelming. Approximately half of the Discussion is in the form of a long first paragraph which only really recaps the Results section, and most of the remaining Discussion is dedicated to the final Caveats paragraph. Overall, this doesn't leave much room for more in-depth discussion.

There are a few possible findings and ideas that could be expanded on in new Discussion paragraphs. I'm not suggesting the authors necessarily need to address all these topics, but I do think the study would be improved with a more thorough discussion of the study's findings and implications for other systems. For example:

- How generalizable should we expect these findings to be for dengue in other countries besides Thailand? Is there anything about Thailand in particular that makes the authors think it should or shouldn't be generalizable to other countries? Has a similar analysis ever been conducted for dengue in other locations?

- How generalizable should these dengue findings be to other infectious diseases? What about other vector-borne diseases that we know are affected by temperature, like malaria? Has a similar analysis ever been conducted for another infectious disease, and if so, what did they find?

- Precipitation is mentioned near the end of the Discussion in relation to what other factors besides temperature may have already affected the patterns of synchrony in the data set. However, in addition to temperature, precipitation is also expected to vary with climate change in the future. Are there any reasons to suspect that this will affect predicted future patterns of synchrony?

Thank you for your comment and suggestion. We have added two paragraphs to the Discussion, one on the generalisability of our results, and another on ENSO (brought up by Reviewer 1, and which also touches on precipitation).

2. Not all readers of this study will have familiarity with time series synchronization analyses. Of course, some knowledge needs to be assumed, but I think defining a few additional terms throughout the manuscript would still help readability. I'll note that I actually think the authors already do a pretty good job of this; for example, when simply describing what WTs are in the first paragraph of the Results, and later what CWMFs are. However, I think there are a few more places additional text/explanation could be included. For example, defining in plain words terms such as the Moran effect, phase angle, wavelet power, and common multiannual fluctuation.

We have added some explanations to the text, as you suggest. We should note that the "common multiannual fluctuation" is not a specific term as such, we were just referring to the fact that we introduced an analogous multiannual fluc-

tuation across locations, so that they all exhibited the same multiannual dynamics at that point in time.

3. As someone who is interested in the mechanistic modeling, I was hoping to see more details on the model in the main text. Indeed, even though lines 162-163 in the Results say to "see Materials and Methods" for the model, there doesn't appear to be any information or subheading regarding the model in the Materials and Methods. I realize a lot of the details are in the supplementary information, but if the authors are not constrained by space I think it would be beneficial to summarize some of that information and potentially the equations themselves under a new subheading in the Materials and Methods section of the main text. I think it would also be useful to explicitly state in the main text how the authors model differs from that of Huber et al., rather than just stating that it builds on it.

We agree with your comment. We were initially concerned with the overall length of the manuscript, and felt that for the interested reader, going to the SI would not have been too much trouble. However, given how central it is to our analyses, adding the model details to the main text does make sense.

We have also clarified the main difference between our model and Huber et al.'s, both in section "Material and methods" (where the model is now presented, following your suggestion), and in section "Mechanistic dengue model and simulations". The difference was limited to the fact that Huber et al.'s model assumed a single serotype (and therefore no interaction between serotypes), whereas ours expands their model to include four serotypes and temporary cross-protection between them.

Minor Comments:

- Line 28: unclear what "predator-prey dynamics between the virus and the host" means. Is this referring to dynamics between the virus and vector? And what type of dynamics?

The reference was predominantly to the phase-shifted fluctuations in the predator (the virus) abundance and the prey (the non-immune host) numbers. We have added this clarification in the text.

- 325-327: This one sentence paragraph seems a bit out of place.

Agreed. We have moved the sentence to the end of the previous paragraph.

- Lines 346-347 note that "During the earlier part of the records, dengue cases might have been detected less effectively, thus potentially obscuring multiannual patterns." Are changes in case detection relevant at any other time in the rest of the time series?

Reporting rates are expected to increase over time (see, e.g., [3]). The key here is whether the reporting is sufficient to qualitatively capture the underlying dynamics. We hypothesise that early on in the records, reporting rates may have been too low to capture the features of the underlying dynamics. Conceivably, beyond some threshold reporting rate, the qualitative elements of the underlying dynamics start being captured. We have tweaked the text in the Discussion to make this clearer.

References

1. Adams B, Holmes EC, Zhang C, Mammen MP, Nimmannitya S, Kalayanarooj S, et al. Cross-protective immunity can account for the alternating epidemic pattern of dengue virus serotypes circulating in Bangkok. Proceedings of the National Academy of Sciences. 2006;103(38):14234–14239. doi:10.1073/pnas.0602768103.

2. Wearing HJ, Rohani P. Ecological and immunological determinants of dengue epidemics. Proceedings of the National Academy of Sciences. 2006;103(31):11802–11807. doi:10.1073/pnas.0602960103.

3. Reich NG, Shrestha S, King AA, Rohani P, Lessler J, Kalayanarooj S, et al. Interactions between serotypes of dengue highlight epidemiological impact of cross-immunity. Journal of the Royal Society Interface. 2013;10(86):20130414. doi:10.1098/rsif.2013.0414.
