# Supplementary material for: Periodic synchronisation of dengue epidemics in Thailand over the last 5 decades driven by temperature and immunity
Source: PLoS Biol. 2022 Mar 18;20(3):e3001160. doi: 10.1371/journal.pbio.3001160 (PMC8967062; doi:10.1371/journal.pbio.3001160)
Supplement: Attachment — Submitted filename: Garcia-Carreras_et_al_response_to_reviewers.pdf [file pbio.3001160.s003.pdf]

Response to reviewers

We would like to thank the reviewer for the comments and suggestions provided, we appreciate them and hope that in addressing these comments (see below) the manuscript is clearer.

We have additionally addressed the Associate Editor's comments:

- *We have added a blurb, as requested.*
- *We have added "between 1968 and 2018" to the title to stress the longitudinal nature of the study. However, we feel that the roles played by temperature and immunity are too complicated to be summarised in a title.*
- *We have edited the abstract to make it more accessible.*
- *We have now included the data underlying all figures as supplementary data files hosted on a github repository.*

Reviewer 1

The paper presents an analysis of the temporal patterns of synchronization of seasonal endemic dengue across space in Thailand. The study is unique in the length of the data set analyzed (5 decades) and the number of spatial locations (72 provinces). The described patterns of localized synchrony with a few episodes of high synchrony/high incidence interspersed among intervals of asynchrony are an interesting finding. The authors also investigate the role of local temperature vs. temperature interacting with immunity, in establishing these patterns. This is done with a process-based model of dengue and its responses to three kinds of forcing: (1) observed temperature, (2) seasonality along a gradient of mean temperatures representative of Thailand (with a pure sinusoidal function), and (3) a single multiannual cycle overlayed on the seasonality. These simulations suggest a role of immunity and its interaction with the extrinsic driver, in particular for the parameters that characterize the more endemic locations. This is not surprising as places with the higher temperatures and therefore, more endemic dengue dynamics, would be the ones where susceptible depletion through patterns of cross-protection play a more important role. Nevertheless, the simulations are valuable to identify parameter ranges/conditions for which this role in interannual variability and therefore, synchrony, would

come into play.

In this revision, the authors have addressed my previous comments and the text is now clear. I have one additional major comment arising from the new figures presented in the Supplement. Although this is only one major concern, it is an important one for the relevance of the simulation results and therefore for the conclusions reached with the model.

Major comment:

Figure S12 now shows the actual time series of reported cases together with the simulated cases produced by the model. If I am not mistaken, comparison of the two temporal trajectories shows a difference of about an order of magnitude in the peaks and temporal mean, with the simulations producing lower infections than those observed in the data. A difference would be expected but in the opposite direction, due to under-reporting. Another way to put it is that the model produces levels of infection that are substantially lower than what is consistent with observations, which is an indication that the parameters of the model are possibly problematic and not representative of the dynamics in this region. I understand and value the effort to use parameters from the literature based on metabolic theory but I wonder to what extent these results indicate that this bottom-up approach, with no calibration at all of the model to the data, cannot really account for the transmission dynamics in the region.

A substantial number of papers has taken a different approach to this question of intrinsic vs. extrinsic factors in the population dynamics of climate-sensitive diseases including vector-borne ones. This approach has been based on methods of parameter inference and model selection, with models fitted to time series. (It may be good to cite at least some of this work as a broader context for the questions here including diseases beyond dengue. See for example, Koelle et al. Nature 2005, or Laneri et al. Plos Comp. Biology 2010, and the many papers that followed).

We have taken on your suggestion and added a sentence in the introduction pointing some of these other studies out:

> *"Much work has gone into disentangling the roles of extrinsic drivers, such as climate, and intrinsic factors in shaping dynamics across many disease systems and using a range of approaches [1, 2, 3, 4, 5, 6]."*

I understand that the approach taken here is different and by design does not involve fitting the model to the data, as such fitting would be challenging for panel data and a disease like dengue with multiple serotypes. Nevertheless,

the simulated cases should be within the plausible range expected for reporting rates in the region, and not as far from the data as what Fig. 12 seems to indicate. This is important for the central argument of the paper because incidence reflects the intensity of transmission, and this intensity should in turn be related to how much of a role immune protection plays in generating multi-annual variation. This issue deserves at least mention and discussion, if not the re-parameterization of the model, unless I am missing something, or the labels of these time series were exchanged in the figure.

We conceived the simulations such that the only element that was different across provinces was the temperature, so all starting conditions, population sizes, birth and death rates, etc., across simulations were the same. We say as much in the caption to Fig S12:

> *"...the model was here run with the same parameters as for all other provinces, i.e., the same total human population size ($1 \cdot 10^5$) and birth and death rates",*

and in section "Further details on simulation studies" in the SI:

> *"...each location was run as an independent simulation (no host movement between locations), using the same host birth and death rates and population sizes and starting conditions ...such that the only difference across locations was temperature".*

This means that the population size for the simulation of dengue in Bangkok is the same as it is in any other province ($1 \cdot 10^5$), which is substantially lower than the actual population of Bangkok. This explains why the numbers produced by the simulation in Fig S12 are lower than the data. However, with these differential equation models, changing the overall population size affects the absolute numbers, but not relative patterns and oscillatory behaviour. Indeed, we normalise all ln-transformed time series to a mean of zero and standard deviation of one prior to performing wavelet transforms. Therefore, our analyses are in no way affected by this mismatch. We agree this could have been made clearer, so have now added the following sentence to section "Further details on simulation studies":

> *"Using the same population sizes across all provinces affects the absolute numbers of infected individuals produced by the model, but not the relative patterns and oscillatory behaviour".*

Thank you for pointing this out.

Minor comments:

1) Abstract: in the third line, the text refers to "climate change". Please change to climate variability and change, as this paper is not primarily about climate

change per se.

Done.

2) Abstract: The sentence "Observed patterns in dengue were statistically more similar. . . ", is somewhat misleading. What is more similar is the pattern of synchrony. (And here comes the problem above, as a reader may interpret this to mean that the model produces patterns of dengue similar to those in the data. For the standards of this kind of work today, this text can give the impression that the models were fitted). Similarly, the sentence "Applying a temperature-driven model. . . ", could be changed to something like "Simulations of a temperature driven model" .

We have now refer to "observed patterns in the synchrony of dengue", and have taken on your suggestion and changed the sentence "Applying a temperature-drive model. . . " to "With simulations from temperature driven models. . . ". Thank you.

3) Lines 111-119: No mention is made here of the difference in the simulations and data concerning whether synchrony is present/absent in early 1970s in Figure 2 . This difference is discussed much later but not acknowledged where first observed.

We make a first brief mention of this discrepancy in section "Driving the model with actual temperature time series", which is where we discuss comparisons between the simulations and data:

> *"Both the WMF and CWMF for the model output contain a notable synchrony event in the early-1970s, reproducing that found in temperature; this feature is absent from the WMF and CWMF estimated using the data (Fig 2b,d)"*

(lines 233–236 in the version of the manuscript you reviewed). Lines 111–119 concern synchrony in temperature only. This discrepancy between data and our model is then more fully addressed in the Discussion.

4) Figures S19-21: adding the scale of the cases in the y-axis would be informative, especially given the possible discrepancy with the observed cases.

As we explain in the response to the main point above, the absolute numbers are not important to the oscillatory behaviour of the model. We feel that adding numbers to the y-axes here would introduce additional text to an already fairly busy figure.

5) Line 335: the correlation is with the patterns of synchrony and not with the observed dengue cases.

Fixed. Thank you.

6) Lines 424-426: here the text touches on the important implications of the work for what one may expect under climate change. It would seem a subject worth expanding on. I did expect that as more places become endemic, more asynchrony rather than synchrony would be expected.

Thank you for pointing this out. We agree that our statement that synchrony may, in the future, be driven by the increasing similarity in temperatures across Thailand is misleading and does not find much support in our results. We have now edited the text to read:

> *"Multiannual cycles may also be, to greater extents, dictated by intrinsic factors over the multiannual timescales present in temperature, meaning that in the future, temperature may be less able to synchronize dengue dynamics."*

However, there are many factors at play that may influence dengue dynamics and synchrony moving forward, making any further statements here highly speculative.

7) Lines 409-412: This statement about dengue is what led to work in the last twenty years on disentangling extrinsic and intrinsic factors in a number of climate-sensitive diseases. Dengue presents the challenge of multiple co-circulating serotypes but it is not unique in this sense, and this question has been a major one in the literature for some time. Worth writing in a way that acknowledges this.

The intent with this paragraph was to specify what features other disease systems might need to have for our approach and work to be useful. However, we agree with this point. We tweaked the start of the sentence you refer to as follows:

> *"The feature that makes dengue a complex system in the context of our study is…",*

thereby hopefully focusing on the "feature", and we then added a sentence to make your point:

> *"This feature is common to other disease systems, and disentangling extrinsic and intrinsic factors has been a major question in previous work [1, 2, 3, 4, 5, 6]."*

References

1. Koelle K, Pascual M. Disentangling extrinsic from intrinsic factors in Disease dynamics: A nonlinear time series approach with an application to cholera. The American Naturalist. 2004;163(6):901–913. doi:10.1086/420798.

2. Koelle K, Rodó X, Pascual M, Yunus M, Mostafa G. Refractory periods and climate forcing in cholera dynamics. Nature. 2005;436(7051):696–700. doi:10.1038/nature03820.

3. Laneri K, Bhadra A, Ionides EL, Bouma M, Dhiman RC, Yadav RS, et al. Forcing versus feedback: Epidemic malaria and monsoon rains in Northwest India. PLOS Computational Biology. 2010;6(9):e1000898. doi:10.1371/journal.pcbi.1000898.

4. Metcalf CJE, Walter KS, Wesolowski A, Buckee CO, Shevliakova E, Tatem AJ, et al. Identifying climate drivers of infectious disease dynamics: recent advances and challenges ahead. Proceedings of the Royal Society B: Biological Sciences. 2017;284(1860):20170901. doi:10.1098/rspb.2017.0901.

5. Paull SH, Horton DE, Ashfaq M, Rastogi D, Kramer LD, Diffenbaugh NS, et al. Drought and immunity determine the intensity of West Nile virus epidemics and climate change impacts. Proceedings of the Royal Society B: Biological Sciences. 2017;284(1848):20162078. doi:10.1098/rspb.2016.2078.

6. Baker RE, Mahmud AS, Wagner CE, Yang W, Pitzer VE, Viboud C, et al. Epidemic dynamics of respiratory syncytial virus in current and future climates. Nature Communications. 2019;10(1):5512. doi:10.1038/s41467-019-13562-y.
