# Supplementary material for: Periodic synchronisation of dengue epidemics in Thailand over the last 5 decades driven by temperature and immunity
Source: PLoS Biol. 2022 Mar 18;20(3):e3001160. doi: 10.1371/journal.pbio.3001160 (PMC8967062; doi:10.1371/journal.pbio.3001160)
Supplement: Attachment — Submitted filename: Garcia-Carreras_et_al_response_to_reviewers.pdf [file pbio.3001160.s005.pdf]

Response to reviewers

Reviewer 1

All my comments have been addressed. In particular, the clarification on population sizes and the changes made concerning population size in the model simulations are useful. I agree with the authors that given the functional form of the transmission term in the equations, the general conclusions and patterns of synchrony should not change because the overall dynamics are independent from population size. Still, the actual number of cases is affected, and seeing these numbers is useful for a comparison to the observed values. This comparison matters because it indicates whether the parameters used in the simulations are consistent with the observed cases. This in turn matters because the parameters determine the importance of immunity (ie the depletion of susceptible individuals), and given that the conclusions concern the role of population immunity, I believe it matters to know whether the parameterization is meaningful for this region. I hope this argument clarifies my reason for requesting the further explanation.

One further request: The difference between the order of magnitude of the observed cases and those simulated should be acknowledged in the Results and discussed in the Discussion, in relation to what is known about the reporting rates in the region and the possible implications for the result (on the importance of immunity). I am not requesting that the model be calibrated or fitted to the data but simply that the difference be recognized and discussed. Ultimately, there is the question of whether models parameterized completely from literature values and published curves for the dependence on temperature are able to represent the dynamics of the disease. This is probably not the case for many reasons. It may not matter for the synchrony patterns but it may influence the interpretation of the results. Now the simulated cases are larger than the observed but by one or two orders of magnitude. Is this an indication of too high a transmission rate? Or is it consistent with reporting rates? The reader would want to know.

The paper is a valuable and interesting study. It would be a shame to have this point "hidden" from discussion.

As requested, we have added text elaborating on the difference between modelled output and data, including the caveat that the discrepancies between the two might be indicative of the model transmission rate possibly being too low. We note, however, that our results were very similar when we had higher R_0 values,

indicating that our conclusions are robust to having higher transmission rates.

We have now added some text to section "Driving the model with actual temperature time series":

> *"The ratios between reported cases and our modelled output (median of 0.11 across all provinces and points in time) are up to almost 30 times greater (for a cross-protection of one year) than those estimated in Reich et al. [1] (median values of between 0.0037–0.0120 across serotypes), suggesting that transmission rates in the model may be comparatively low. However, our results were robust to changes in transmission (see section "Robustness tests" in SI).",*

and to the Discussion:

> *"The mechanistic model we use here, when compared to data, also leads to reporting ratios that are distinctly higher than those estimated in the literature [1], implying transmission rates in the model may be low. Lower transmission rates might be expected to affect the dynamics of immunity, as susceptibles would be depleted at a slower rate, and would lead to an increase in the periodicity of multiannual cycles produced by immunity. Nonetheless, our results on synchrony are robust to variations in transmission."*

References

1. Reich NG, Shrestha S, King AA, Rohani P, Lessler J, Kalayanarooj S, et al. Interactions between serotypes of dengue highlight epidemiological impact of cross-immunity. Journal of the Royal Society Interface. 2013;10(86):20130414. doi:10.1098/rsif.2013.0414.
